# Effect of chiral 2-ethylhexyl side chains on chiroptical properties of the narrow bandgap conjugated polymers PCPDTBT and PCDTPT†
†Electronic supplementary information (ESI) available: Detailed synthetic procedures, CDA NMR spectra, DSC curves, additional UV-Vis and CD spectra, AFM images, GIWAXS line cuts, DFT calculation details. See DOI: 10.1039/c6sc00908e


**DOI:** 10.1039/c6sc00908e

**Published:** 2016-05-03

**Authors:** Stephanie L. Fronk, Ming Wang, Michael Ford, Jessica Coughlin, Cheng-Kang Mai, Guillermo C. Bazan

**Affiliations:** a Center for Polymers and Organic Solids , University of California , Santa Barbara , California 93106 , USA . Email: bazan@chem.ucsb.edu; b Department of Chemistry and Biochemistry , University of California , Santa Barbara , California 93106 , USA; c Materials Department , University of California , Santa Barbara , California 93106 , USA; d King Abdulaziz University , Jeddah 21413 , Saudi Arabia

## Abstract

Two narrow bandgap conjugated polymers containing chiral 2-ethylhexyl side chains were synthesized: poly[(4,4-bis(2-ethylhexyl)cyclopenta-[2,1-*b*:3,4-*b*′]dithiophene)-2,6-diyl-*alt*-(2,1,3-benzothiadiazole)-4,7-diyl] (PCPDTBT*) and poly[(4,4-bis(2-ethylhexyl)cyclopenta[2,1-*b*:3,4-*b*0]dithiophene)-2,6-diyl-*alt*-[1,2,5]-thiadiazolo[3,4-*c*]pyridine] (PCDTPT*). The presence of a chiral substituent provides a handle to study the geometry of interchain aggregates and/or the secondary structure of these conjugated polymers in solution and in thin films *via* circular dichroism (CD) spectroscopy, provided that the asymmetry in the side-chain is translated to the optically active conjugated backbone. CD signals are observed for PCPDTBT* and PCDTPT* in poor solvent systems, which indicate the presence of chiral ordering in the aggregates. PCPDTBT* shows greater chiral order than PCDTPT* based on their relative anisotropy factors. Additionally, GIWAXS analysis reveals that PCPDTBT* films are more ordered than what is observed for the same polymer containing racemic 2-ethylhexyl chains. Upon solution deposition, the chiral ordering is found to translate to the solid-state microstructure for PCPDTBT* but not PCDTPT*. The presence of a pyridyl nitrogen on the thiadiazolo[3,4-*c*]pyridine ring of PCDTPT* favors a planar conformation for the backbone such that it has a higher rotational barrier compared to PCPDTBT*. This larger rotational barrier appears to limit the ability of PCDTPT* to adopt a helical structure or relevant chain distortions for achieving chiral aggregates.

## Abstract 2

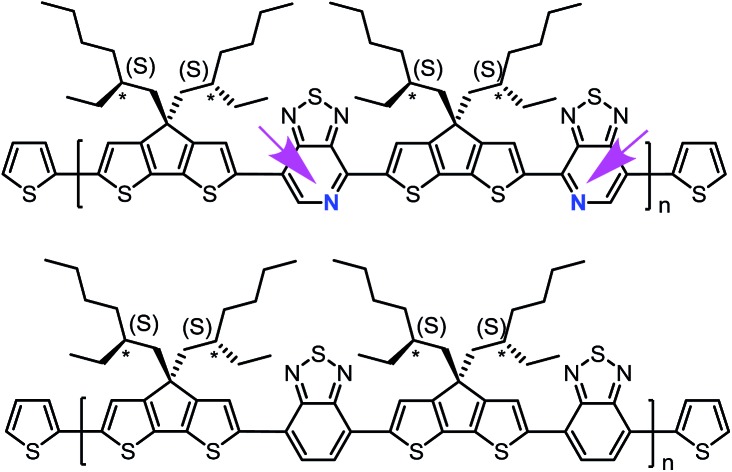
PCPDTBT* and PCDTPT* containing chiral 2-ethylhexyl side chains were synthesized and their resulting chiroptical properties were studied. PCPDTBT* exhibits a stronger chiroptical response compared to PCDTPT*.

## Introduction

Narrow bandgap conjugated polymers based on alternating electron rich (D) and electron poor (A) structural units have attracted extraordinary interest due to their use as the semiconducting component in solution fabricated organic solar cells^1^ and thin film transistors.^2^ Achieving ordered polymer domains in the thin film is typically required to obtain more effective electronic coupling and appropriate registry between electronically delocalized subunits.^3^ Improved electronic coupling ultimately leads to higher charge carrier mobilities; an important parameter that mediates charge extraction in solar cells and the velocity of charge carriers in transistor channels.^4^ Side chains that improve the solubility of the backbone are essential elements for the design of the overall polymer molecular structure, since these groups mediate interchain contacts in the solid state, thermal transitions in the bulk, and the orientation of aromatic segments relative to the substrate.^5^ Moreover, the choice of side chain can be used to control the degree of interchain aggregation in solution and therefore important intermediates in the transition from isotropic solvated chains to the organization in the bulk.^6^

More detailed insights into the states of the narrow bandgap conjugated polymer chains in solution and in particular those that may be present transiently during the transition to the solid state would be helpful to better predict the optimal processing conditions that achieve desirable device characteristics.^7^ Little emphasis has been placed on understanding preferred secondary structures as a function of backbone structural units and how these topological features impact collective behavior.^8^

Relevant related work has centered on conjugated polymers with chiral side chains, since the resulting chiroptical properties determined by using circularly polarized light yield information on the driving force for distortions that produce a chiral backbone chromophore.^9^ Literature examples exist on systems based on polythiophene,^10^ polyacetylene,^11^ poly(phenylenevinylene),^12^ and polyfluorenes.^13^ These studies have demonstrated how introducing chirality provides opportunities for self-assembly into novel supramolecular structures and for attaining control over the solid state microstructure.^14^ The chiroptical properties of narrow bandgap conjugated polymers, however, have received limited attention.^15^

The 2-ethylhexyl substituent has been widely used to solubilize conjugated polymers. Although this fragment has a chiral center, it has been overwhelmingly used in its racemic form. In this contribution, we focus on the design, synthesis, and physical properties of two narrow bandgap conjugated polymers with chiral 2-ethylhexyl substituents. One structure is a derivative of poly[(4,4-bis(2-ethylhexyl)cyclopenta-[2,1-*b*:3,4-*b*0]dithiophene)-2,6-diyl-*alt*-(2,1,3-benzothiadiazole)-4,7-diyl] (PCPDTBT). PCPDTBT has been extensively studied as a donor material for the fabrication of bulk heterojunction solar cells that contain soluble fullerene derivatives as the acceptor component.^16^ The second polymer to investigate contains cyclopenta[2,1-*b*:3,4-*b*′]dithiophene (CDT) and pyridyl[2,1,3]thiadiazole (PT) structural units arranged along the polymer backbone in a strict regioregular fashion. Aligned polymers with these repeat units exhibit exceptional hole transport in organic field effect transistors (OFETs).^17^ Specifically, we describe a derivative with 2-ethylhexyl groups, namely poly[(4,4-bis(2-ethylhexyl)cyclopenta[2,1-*b*:3,4-*b*0]dithiophene)-2,6-diyl-*alt*-[1,2,5]-thiadiazolo[3,4-*c*]pyridine] (PCDTPT). Both polymers designated here as PCPDTBT and PCDTPT rely on the 2-ethylhexyl groups to promote solubility in organic solvents.

**Fig. 1:**
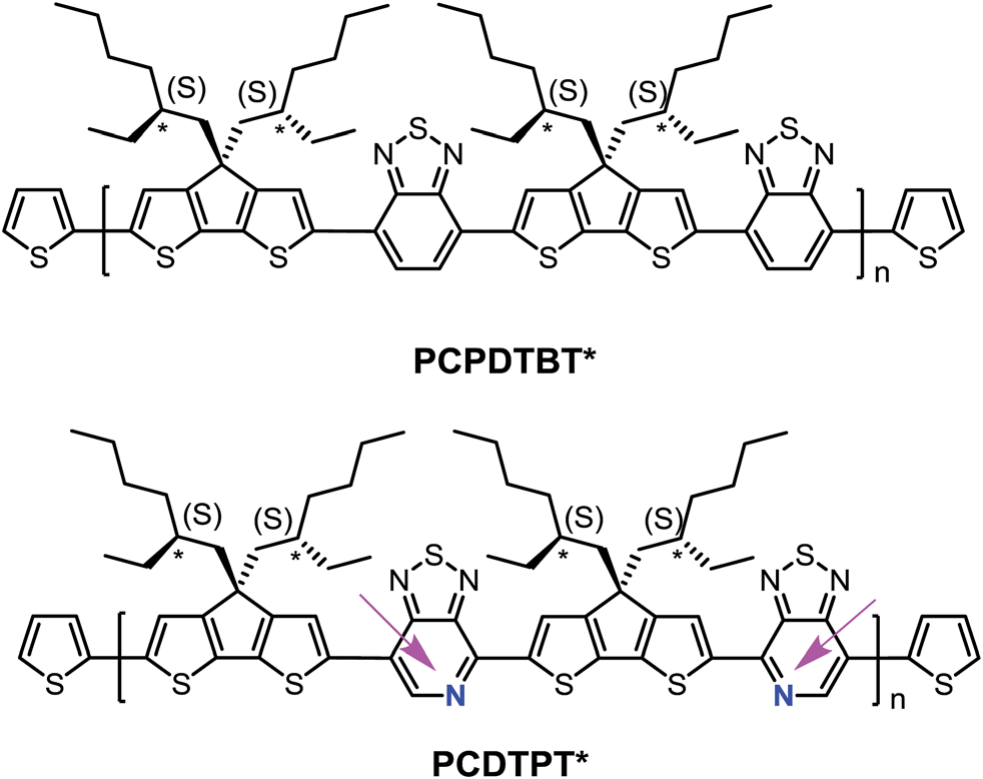
Molecular structures of PCPDTBT* and PCDTPT*. The arrows are meant to guide the eye and highlight the regioregularity of the PT units relative to the backbone direction.

Structural substitution of racemic (PCPDTBT and PCDTPT) *vs.* chiral (PCPDTBT* and PCDTPT*, see Fig. 1) 2-ethylhexyl fragments introduces the possibility of examining chiroptical response, provided that the asymmetry of the side chains is translated to the conjugated backbone chromophore. Chiral macromolecules are known to form chiral nanostructures either as individual macromolecules, such as helices, or as aggregates.^18^ Thus, substitution of chiral for racemic side chains opens the possibility to explore the coil conformations of the polymers resulting from the asymmetry of the side chains. Note that the substitution of a pyridyl nitrogen for C–H on the benzothiadiazole unit is the only structural difference between the two backbones. By comparing the chiroptical responses of PCPDTBT *vs.* PCPDTBT* and PCDTPT *vs.* PCDTPT*, we gain insight into the influence of the pyridyl nitrogen on the secondary structure or the geometry of multichain aggregates of the polymer. We find that PCPDTBT* and PCDTPT* produce a chiroptical response only when aggregated in solution. This asymmetry is translated to the solid state only in the case of PCPDTBT*. These differences are discussed within the context of how rotational barriers may influence distortions needed to achieve helical structures.

## Results and discussion

### Synthesis

**Scheme 1:**
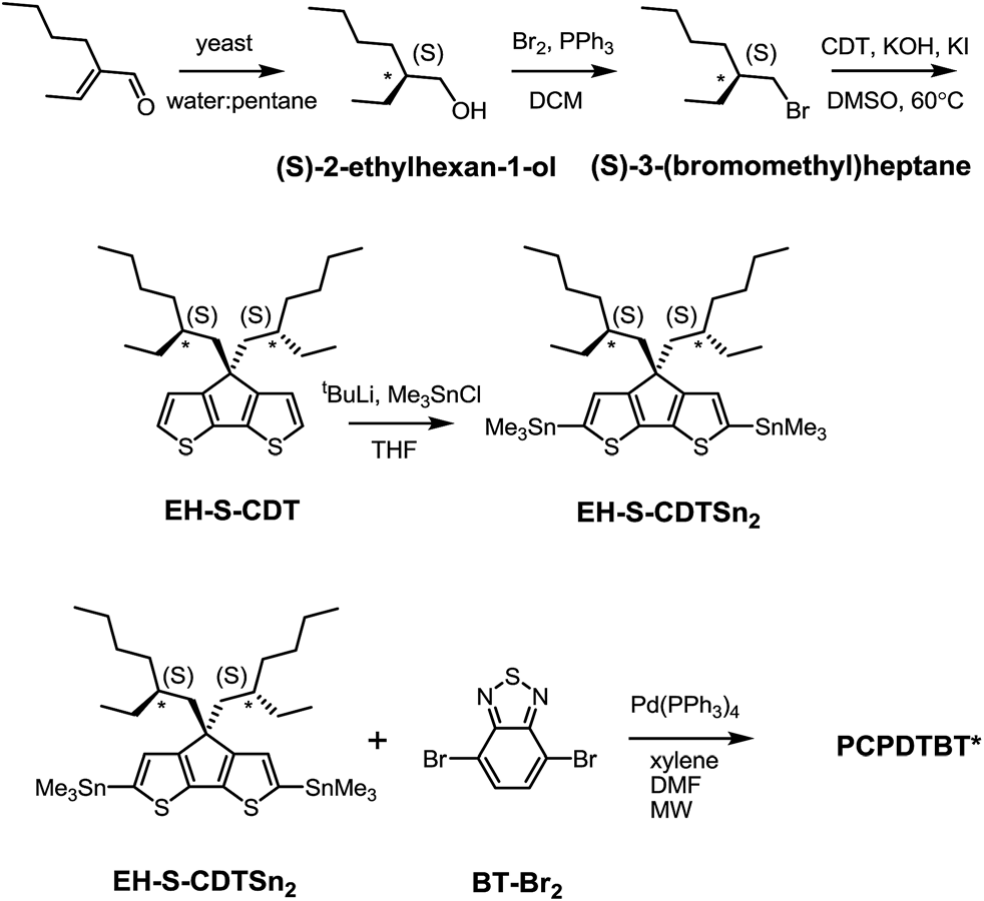
Synthesis of PCPDTBT*.

**Scheme 2:**
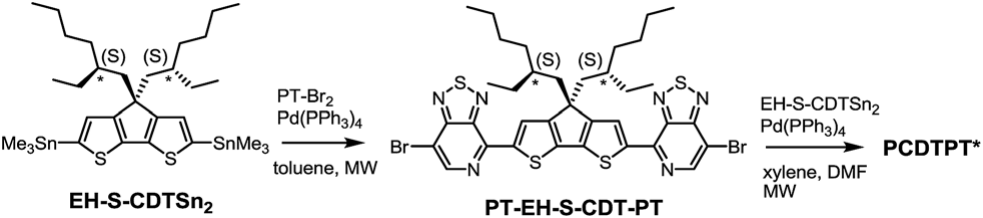
Synthesis of PCDTPT*.

As shown in Scheme 1, (*S*)-2-ethylhexan-1-ol was synthesized according to literature procedures.^19^ Reduction of the α,β-unsaturated aldehyde to the chiral alcohol is accomplished using Baker's yeast as a biocatalyst. High chiral purity was deemed necessary to accentuate any changes in polymer structural characteristics. The optical purity of (*S*)-2-ethylhexan-1-ol was confirmed using polarimetry and through the use of a chiral derivatizing agent (CDA). The optical rotation was found to be +2.95 when measured at a concentration of 1.2 g/100 mL in acetone and the enantiomeric excess was determined to be 95% based upon comparison with the known optical rotation.^20^ CDAs are used to determine stereochemistry and chiral purity using Mosher's method.^21^ Diastereomers have different ^1^H NMR chemical shifts, whereas enantiomers do not; thus a reaction to form a diastereomer allows for determination of enantiomeric purity.^22^ Accordingly, (*S*)-2-ethylhexan-1-ol was reacted with (*S*)-(+)-10-camphorsulfonyl chloride (Scheme S2†) to produce an adduct with a distinct doublet peak at 4.16 ppm (Fig. S1a†). In contrast, the corresponding analogous adduct resulting from the reaction with racemic 2-ethylhexan-1-ol (Scheme S3†) gave rise to a multiplet of peaks at 4.2–4.1 ppm (Fig. S1b†). These CDA results confirm the high chiral purity of the 2-ethylhexyl framework subsequently used to solubilize the polymer chains.

The remaining sequence of steps required for the preparation of the target PCPDTBT* is outlined in Scheme 1. (*S*)-2-ethylhexan-1-ol was converted to (*S*)-3-(bromomethyl)heptane *via* reaction with 2.7 eq. Br_2_ and 1.4 eq. PPh_3_ in dichloromethane. Attachment of the chiral side groups to the CDT framework to yield EH-S-CDT was accomplished by employing previously described alkylation protocols.^23^ Reaction of EH-S-CDT with 2.5 eq. ^*t*^BuLi and 3 eq. Me_3_SnCl provided the bis-stannylated intermediate EH-S-CDTSn_2_. The polymerization between EH-S-CDTSn_2_ (1.05 eq.) and 4,7-dibromo-2,1,3-benzothiadiazole (BT-Br_2_) (1 eq.) was carried out by using microwave-assisted Stille-coupling polymerization conditions in a xylene:DMF solvent system with 0.052 eq. Pd(PPh_3_)_4_ as the catalyst.16*a* After purification using Soxhlet extraction, the resulting polymer, PCPDTBT*, was obtained with a number average molecular weight (*M*_n_) of 23 K and a dispersity (*Đ*) of 1.6. The analogous polymer with racemic 2-ethylhexyl side chains (PCPDTBT) was also prepared in order to have a reference material. This procedure follows literature precedent and can be found in the ESI (Scheme S10†). PCPDTBT was obtained with *M*_n_ = 32 K and *Đ* = 2.3. Characterization by differential scanning calorimetry (DSC) reveals no phase transitions between 20 °C and 300 °C for either material (Fig. S2†), in agreement with previous studies on PCPDTBT.16*a*

PCDTPT* was synthesized as shown in Scheme 2.17*a* A regiospecific Stille coupling reaction between EH-S-CDTSn_2_ and two equivalents of PT-Br_2_ was used to provide PT-EH-S-CDT-PT. The target polymer was attained through the reaction of 1 eq. PT-EH-S-CDT-PT and 1.05 eq. EH-S-CDTSn_2_ and subsequent purification *via* Soxhlet extraction. PCDTPT* obtained in this manner had *M*_n_ = 17 K and *Đ* = 1.7. The analogous reference polymer with racemic 2-ethylhexyl side chains (PCDTPT, *M*_n_ = 23 K and *Đ* = 2.0) was synthesized using the same procedure (Scheme S11†). Characterization by DSC shows no phase transitions between 20 °C and 300 °C for either material (Fig. S3†).

### Absorption and circular dichroism spectroscopies of PCDPDTBT and PCPDTBT* in different solvents

Optical absorption and circular dichroism (CD) spectra were measured for PCPDTBT* and PCPDTBT in dilute chlorobenzene (CB) and in a 1 : 1 CB : diiodooctane (DIO) solvent mixture. DIO is known to be a poor solvent for PCPDTBT and has been used as a solvent additive for modifying bulk heterojunction PCPDTBT/fullerene blend morphologies during the spin-coating deposition step.^24^ A solvent ratio of 1 : 1 CB : DIO was chosen for this study as aggregation of polymer chains is favored for PCPDTBT* and PCPDTBT without causing either material to obviously precipitate out of solution. Specifically, PCPDTBT is known to form aggregates as the volume of DIO is increased relative to CB.^25^

**Fig. 2:**
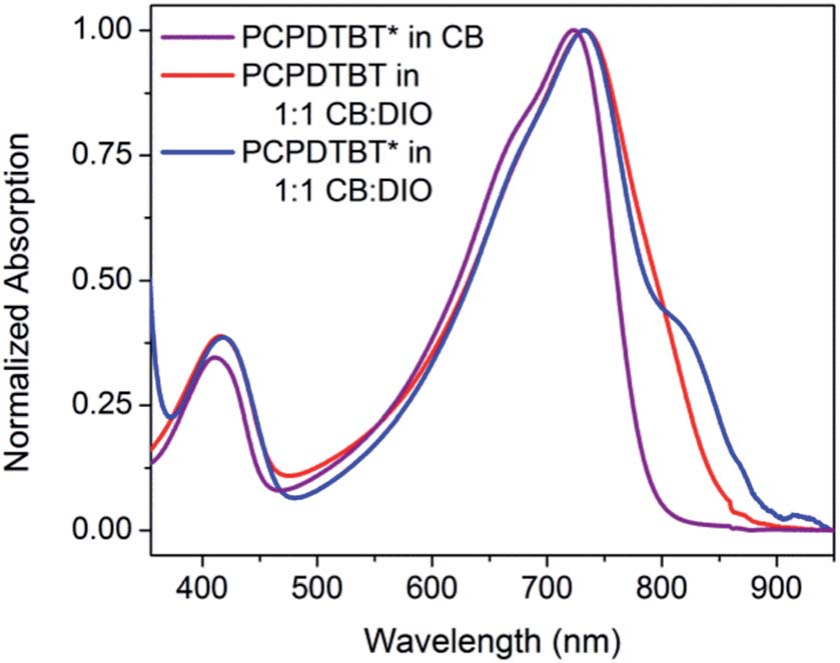
Normalized optical absorption spectra of PCPDTBT* in CB (purple), PCPDTBT in CB : DIO (red), and PCPDTBT* in CB : DIO (blue).

The optical absorption spectrum of PCPDTBT* in dilute CB shown in Fig. 2 is consistent with a solvated polymer in a good solvent with minimal interchain interactions.16*a* Evidence for aggregate formation is observed for PCPDTBT* in CB : DIO. Specifically, PCPDTBT* shows a distinct shoulder peak at longer wavelengths (795–810 nm). This feature has been previously implicated to arise from aggregated chains in solution^25^ and is more prominent for PCPDTBT* than PCPDTBT, as determined by the further red shift of this shoulder and better defined features. Further evidence of greater order within PCPDTBT* aggregates is provided by the analysis of thin films, as discussed in the next section. Note also that the *M*_n_ of PCPDTBT* is lower than that of PCPDTBT, which should discourage aggregation. Altogether, these observations indicate preferential aggregation of PCPDTBT* as a result of the greater structural precision of the solubilizing side chains.

**Fig. 3:**
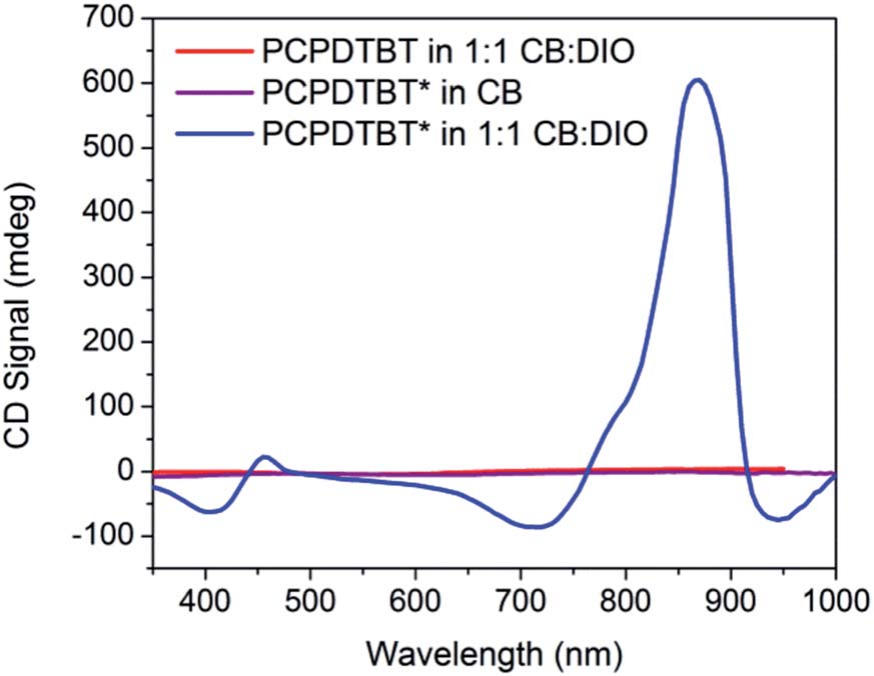
CD spectra of PCPDTBT* in CB (purple), PCPDTBT in CB : DIO (red), and PCPDTBT* in CB : DIO (blue).

Fig. 3 provides the CD spectra for PCPDTBT* and PCPDTBT as a function of solvent conditions. These data reveal that CD signals are present for PCPDTBT* in CB : DIO, but not for PCPDTBT* in CB or for PCPDTBT in CB : DIO. A bisignate Cotton effect is observed for PCPDTBT* at the higher energy π → π* transition. There is also a CD signal at longer wavelengths (centered at 870 nm), primarily located within the absorption profile of the aggregate. This result, combined with the absence of a CD signal in CB solution, supports the hypothesis that PCPDTBT* is chiroptically active only when the chains are incorporated within a multichain aggregate.12*c* The CD signal at 950 nm, where the optical absorption is negligible is most likely due to scattering by the aggregates.

**Fig. 4:**
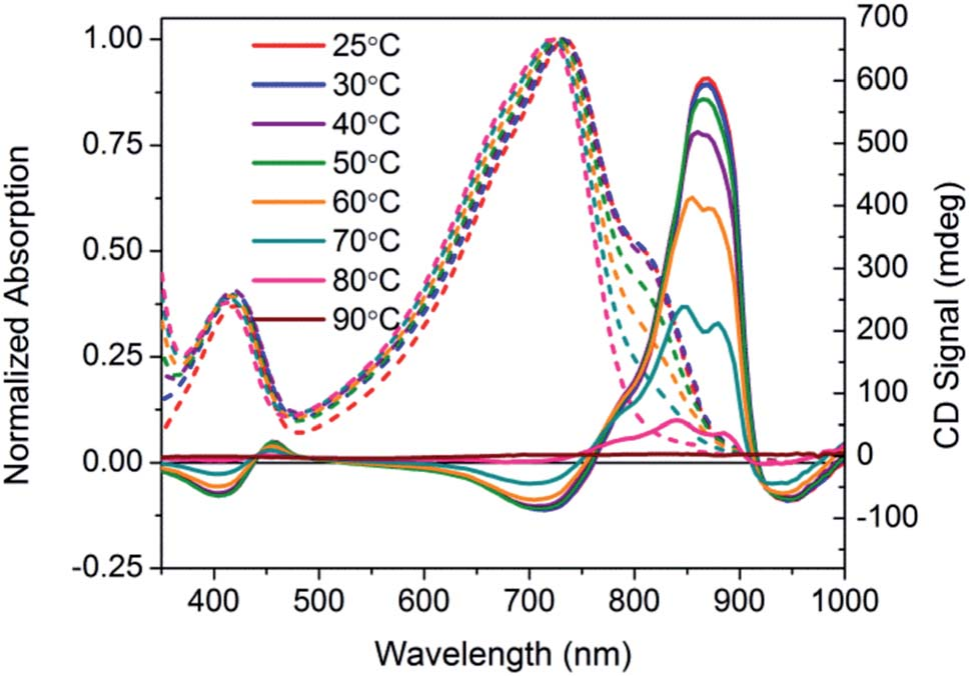
Normalized optical absorption (dashed) and CD (solid) spectra of PCPDTBT* in CB : DIO as a function of temperature, from 25–90 °C.

Temperature dependent spectra of PCPDTBT* were measured in dilute CB : DIO solutions, and the results are shown in Fig. 4. In these experiments, solutions were heated from 25 °C to 90 °C and absorption/CD measurements were taken every 10 °C. Absorption measurements show a progressive decrease in the shoulder peak attributed to aggregates with increasing temperature. Such a process is reasonable; the solubility of the chains improves thereby increasing the fraction of the sample present as well-solvated chains with less pronounced interchain contacts. Once 80 °C is reached, the shoulder peak disappears and the spectrum resembles that obtained in a good solvent where polymer chains are well-dissolved. Temperature dependent absorption of PCPDTBT was also measured and shows a similar decrease in shoulder peak with increasing temperature (Fig. S6†). The CD signal also decreases with increasing temperature. By 90 °C, no CD signal was observed. This correspondence between thermochromic behavior and CD signatures further confirms that chiroptical properties depend upon interchain interactions.

**Fig. 5:**
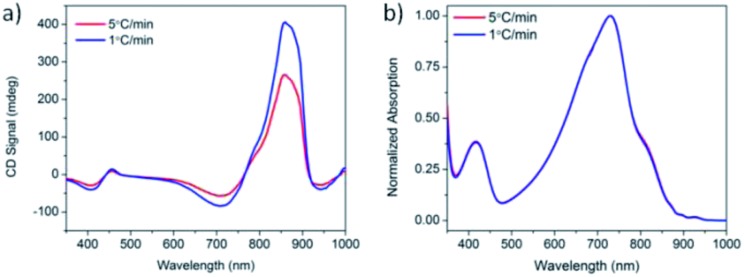
(a) CD and (b) normalized absorption spectra of PCPDTBT* in CB : DIO measured 1 week after cooling at 5 °C min^–1^ (red) and 1 °C min^–1^ (blue).

Solutions of PCPDTBT* in CB : DIO were heated to 90 °C, held at 90 °C for 15 minutes, and cooled back to 25 °C at cooling rates of 1 °C min^–1^ and 5 °C min^–1^. One week after performing these controlled cooling measurements, the CD response was again measured (Fig. 5a). The solution cooled at 1 °C min^–1^ displayed a stronger CD signal at the aggregate absorption peak (centered at 870 nm), compared to the solution cooled at 5 °C min^–1^. However, the absorption profile of the two solutions remains the same (Fig. 5b). Varying the cooling rate therefore does not change the aggregate absorption peak. This suggests the difference in CD signal stems from different order within the aggregate rather than a different ratio of aggregated and solvated chains. Cooling at a slower rate allows the polymer chains to reach a more thermodynamically-preferred structure, whereas cooling at a faster rate leaves the polymer chains kinetically trapped in a less well-ordered state.

The degree of circular polarization in absorption can be estimated using the anisotropy factor, *g*_abs_, which compares the difference in left (*A*_L_) and right (*A*_R_) circularly polarized light with the linear optical absorption (*A*) (*g*_abs_ = (*A*_L_ – *A*_R_)/*A*).^26^ The average *g*_abs_ calculated for the aggregate absorption peak in CB : DIO (825 nm) of PCPDTBT* is 0.013.

### Solid state characterization of PCPDTBT*

**Fig. 6:**
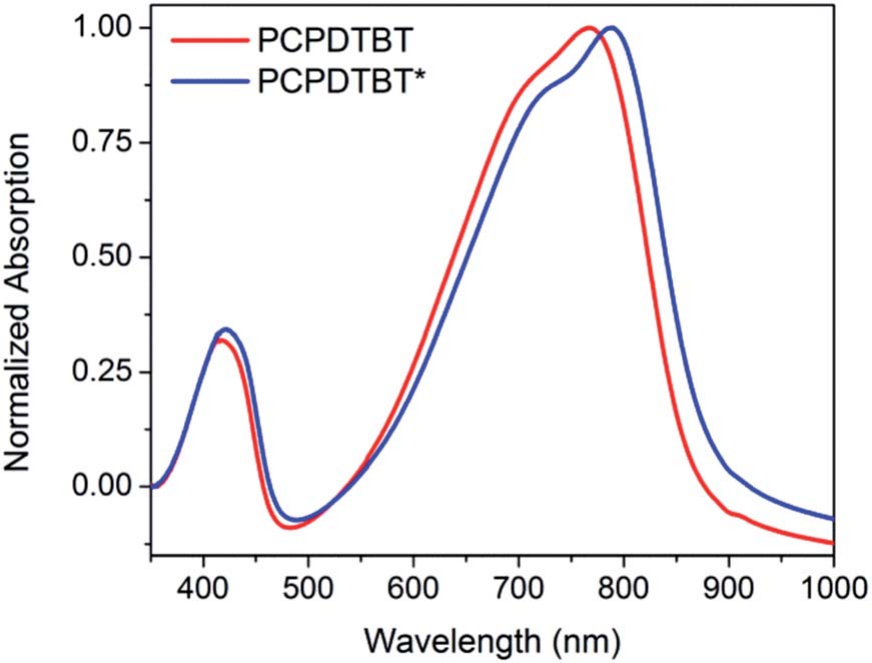
Normalized thin film absorption spectra of PCPDTBT (red) and PCPDTBT* (blue).

Thin films of PCPDTBT* and PCPDTBT were obtained by spin-coating 8 mg mL^–1^ CB solutions onto clean glass substrates and were examined using optical absorption spectroscopy, CD spectroscopy, atomic force microscopy (AFM), and grazing incident wide-angle X-ray scattering (GIWAXS). Optical absorption spectroscopy of both materials shows a red shift in absorption toward the low energy shoulder peak observed in solution (Fig. 6). The PCPDTBT absorption spectrum agrees with the literature.16*a* However, PCPDTBT* shows a small redshift in absorption maximum (21 nm) compared to PCPDTBT.

**Fig. 7:**
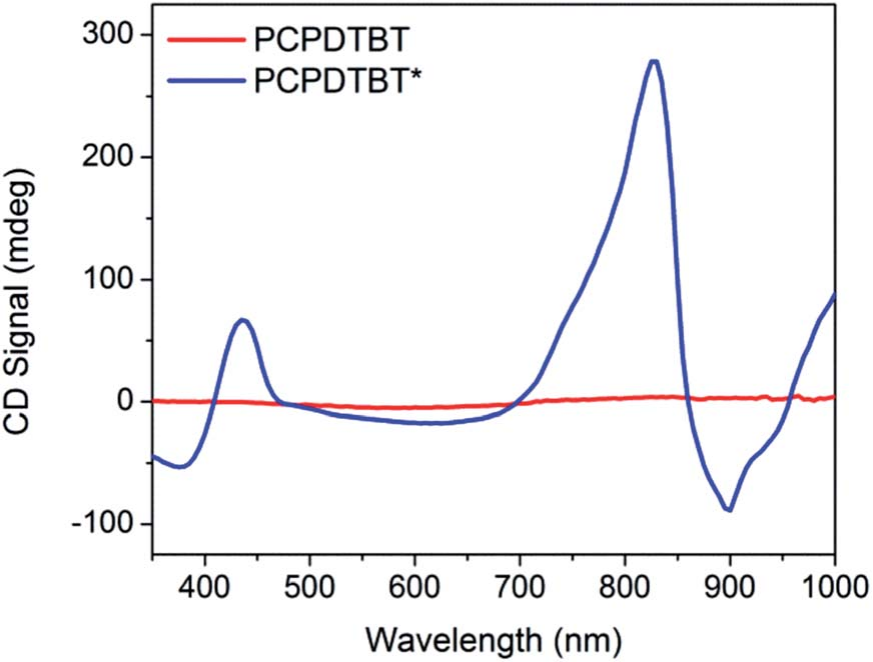
Thin film CD spectra of PCPDTBT (red) and PCPDTBT* (blue).

Thin film CD spectra are shown in Fig. 7. PCPDTBT thin films show no chiral response, as expected. PCPDTBT* thin films show two CD signatures similar to those observed in solution. A clear bisignate Cotton effect is observed at the high-energy π → π* transition. This CD peak is more prominent in the thin film compared to solution. A bisignate Cotton effect is also observed at the absorption aggregate peak (centered at 825 nm). No significant change in absorption or CD signal is observed when films are thermally annealed (Fig. S7 and S13†).

The anisotropy factor can also be calculated for thin films. The average *g*_abs_ calculated for the aggregate absorption peak (830 nm) of PCPDTBT* is 0.037. Therefore, the *g*_abs_ value increases for the thin film compared to solution.

**Fig. 8:**
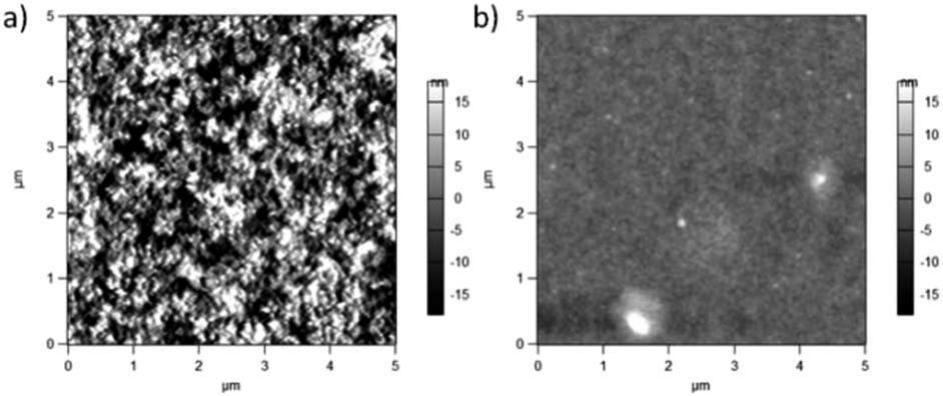
AFM topography images (5 × 5 μm^2^) of (a) PCPDTBT* and (b) PCPDTBT thin films.

Possible changes in surface features were studied using AFM. Surface topography images are shown in Fig. 8. PCPDTBT shows a smooth film, whereas PCPDTBT* shows fiber-like structure. The surface roughness of PCPDTBT* (8.5 nm) is also greater than that of PCPDTBT (2.8 nm). These results provide the first indication of a difference in solid state ordering induced by chiral *vs.* racemic side chains.

**Fig. 9:**
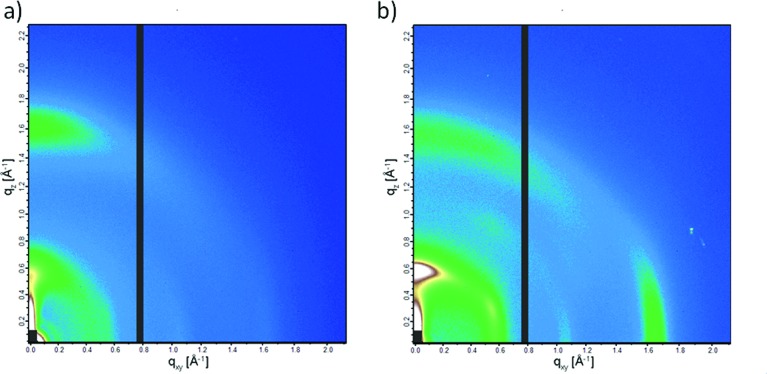
2-D GIWAXS images of (a) PCPDTBT and (b) PCPDTBT*.

Crystalline domains in the thin films of PCPDTBT* and PCPDTBT were studied using GIWAXS.^27^ The 2-D GIWAXS images are shown in Fig. 9 and line cut profiles are shown in Fig. S17.† The π–π stacking peaks are observed at 16.7 nm^–1^ (*d* = 0.38 nm) and 15.8 nm^–1^ (*d* = 0.40 nm) for PCPDTBT* (Fig. 9b) and PCPDTBT (Fig. 9a), respectively. The alkyl chain stacking peaks are observed at 5.87 nm^–1^ (*d* = 1.07 nm) and 5.51 nm^–1^ (*d* = 1.14 nm) for PCPDTBT* and PCPDTBT respectively, which is in agreement with the literature for PCPDTBT.^28^ The increase in *d*-spacing with the presence of the racemic groups suggests that PCPDTBT* exhibits tighter packing compared to PCPDTBT. Crystalline correlation lengths (CCL) were calculated for both films using the Scherrer equation.^29^ The CCL for the alkyl chain stacking peak of PCPDTBT is 2.05 nm. The CCL value for PCPDTBT* is 3.78 nm, almost double that of PCPDTBT. The CCLs for the π–π stacking are 8.10 nm for PCPDTBT* and 6.24 nm for PCPDTBT. The increase in the CCL for the chiral polymer indicates larger or more perfect crystallites with increasing homogeneity of molecular structure.

For equally thick films with isostructural materials, the GIWAXS diffraction intensity can be related to the amount of crystalline component in the film.^27^ The 2-D GIWAXS image of PCPDTBT* displays an intense peak at 16.7 nm^–1^ in the in-plane direction which is almost absent in PCPDTBT. Similarly, the scattering peak intensity of the alkyl chain stacking peak in the out-of-plane direction is much stronger for PCPDTBT* than PCPDTBT. The greater scattering intensity of PCPDTBT* films compared to PCPDTBT suggests a larger fraction of crystalline material in PCPDTBT* films. Combining the CCL and scattering intensity data, we find that PCPDTBT* films are substantially more ordered than PCPDTBT films.

There exist several possible intramolecular conformations and intermolecular ensemble arrangements for polymers with chiral side chains that can give rise to CD signals.^14^,^30^ Single polymer chains may form helical secondary structures while remaining well-dissolved in solution or when introduced into confined environments. Alternatively, asymmetric supramolecular structures may form, analogous to cholesteric liquid crystals, in which there is no requirement for a non-planar polymer backbone. Fully solvated PCPDTBT* is unlikely to form a stable helical structure based on the absence of a CD signal in a good solvent. Differentiating between a helical backbone structure within the aggregates and a supramolecular chiral packing motif is more difficult. Moreover, there is no reason why chiral helical chains cannot pack in non-centrosymmetric units.

### Chiroptical solution characterization of PCDTPT*

The chiroptical properties of PCPDTBT* provoke the question of generality in structurally related conjugated polymers. PCDTPT* was therefore synthesized and characterized for comparison. The mixed solvent system tested with PCPDTBT* (1 : 1 CB : DIO), did not induce aggregation of PCDTPT chains. Therefore, multiple solvent systems were screened and 8 : 2 CB : dimethyl sulfoxide (DMSO) showed the optimal balance of inducing aggregation without causing the polymer chains to precipitate out of solution.

**Fig. 10:**
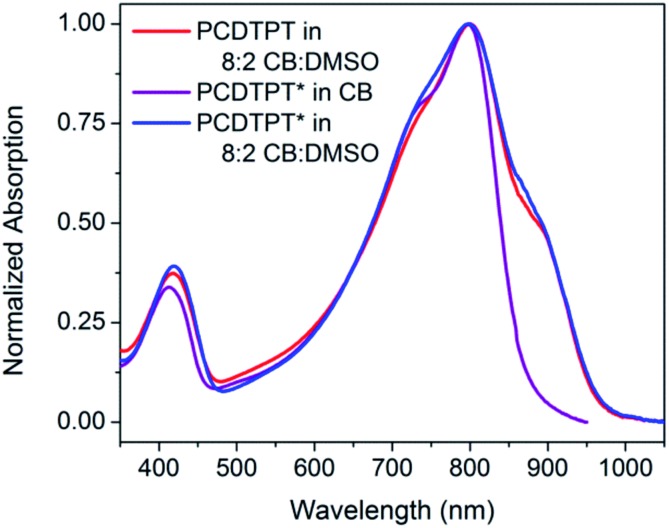
Normalized optical absorption spectra of PCDTPT in CB : DMSO (red), PCDTPT* in CB (purple), and PCDTPT* in CB : DMSO (blue).

**Fig. 11:**
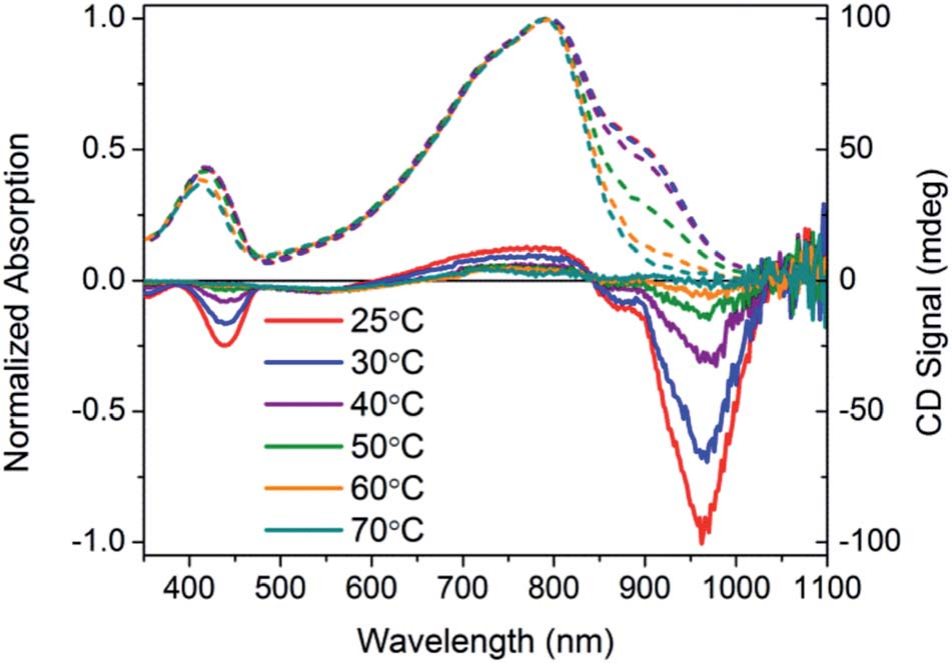
Normalized optical absorption (dashed) and CD (solid) spectra of PCDTPT* in CB : DMSO as a function of temperature, from 25–90 °C.

As shown in Fig. 10, dilute CB : DMSO solutions of PCDTPT* and PCDTPT show very similar absorption profiles, with shoulder peaks at ∼875 nm. The spectra obtained in the poor solvent mixture is substantially red-shifted from what is observed for PCDPTP* in the good solvent CB. As the temperature is increased, the absorption shoulder of PCDTPT* in CB : DMSO progressively disappears (Fig. 11). These features qualitatively resemble the temperature dependence of PCPDTBT* absorption.

**Fig. 12:**
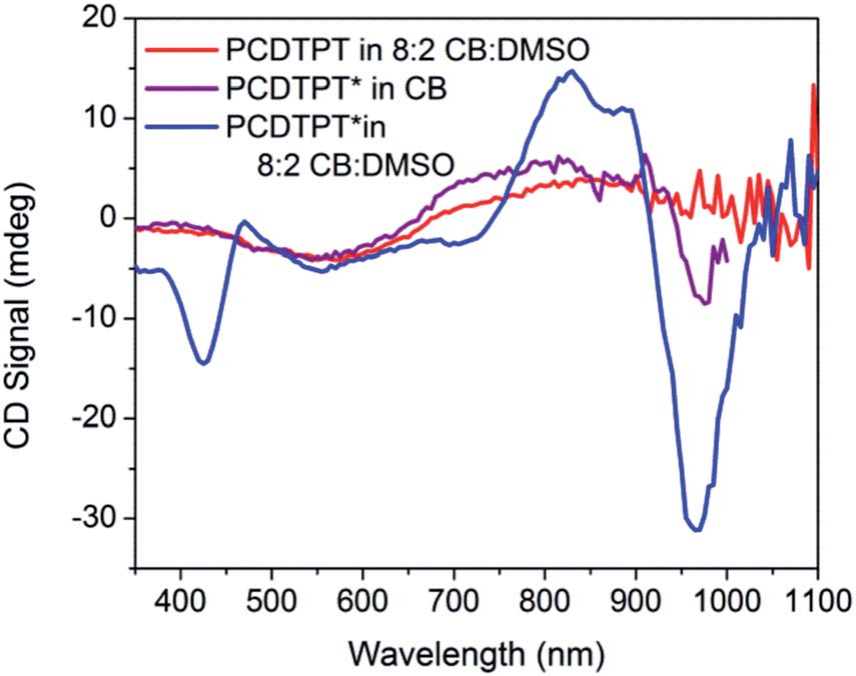
CD spectra of PCDTPT in CB : DMSO (red), PCDTPT* in CB (purple), and PCDTPT* in CB : DMSO (blue).

CD signals are present for PCDTPT* in CB : DMSO solutions but not for PCDTPT in CB : DMSO or PCDTPT* in CB solutions (Fig. 12). Similar to PCPDTBT*, a bisignate Cotton effect is observed at the high energy π → π* transition. A second bisignate Cotton effect is centered near the absorption shoulder peak (960 nm). The CD spectrum of PCDTPT* as an aggregate in solution is opposite in sign compared to that of PCPDTBT* despite containing the same chiral substituent. A plausible explanation for this phenomenon is the different solvent system used. Previous work has highlighted how a change in solvent polarity can induce an inversion of the CD spectrum for a single material.9a,10d,^31^ DMSO is a more polar solvent than DIO, potentially giving rise to a similar effect in this study. As solutions of PCDTPT* in CB : DMSO are heated, the CD signal essentially disappears (Fig. 11). These data further confirm the necessity of a multichain aggregate to achieve chiroptical response. For PCDTPT* in CB : DMSO, the average *g*_abs_ calculated for the aggregate absorption peak (920 nm) is –0.0016, much smaller in magnitude relative to PCPDTBT* in CB : DIO (0.013).

### Solid state characterization of PCDTPT*

**Fig. 13:**
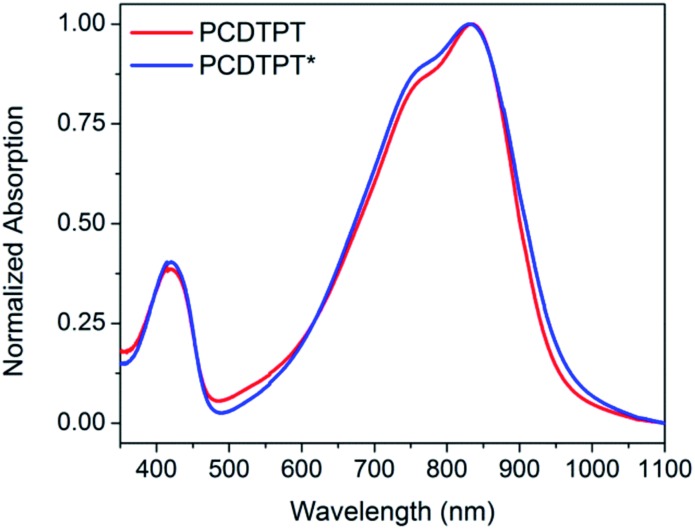
Normalized thin film absorption spectra of PCDTPT (red) and PCDTPT* (blue).

**Fig. 14:**
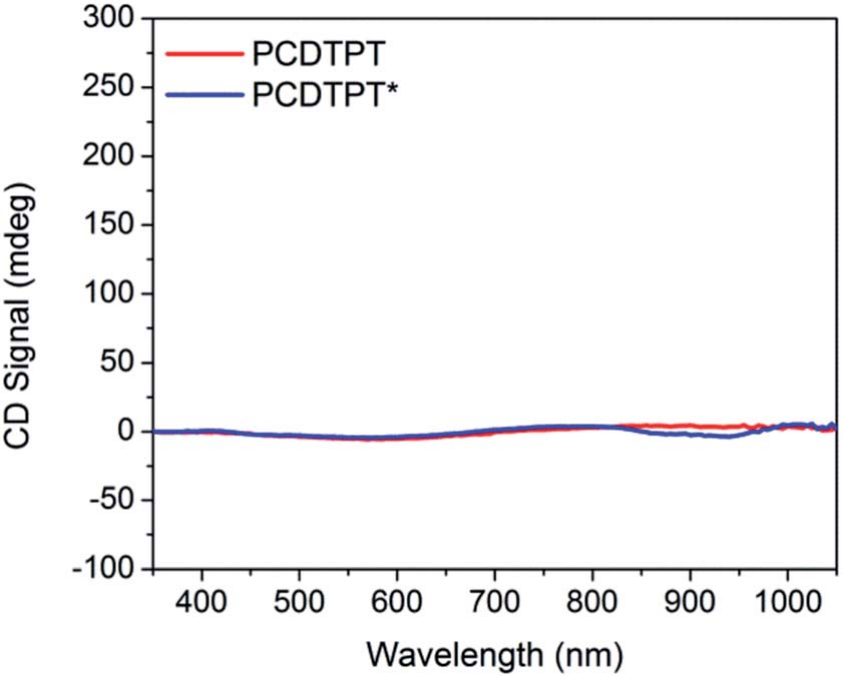
CD spectra of thin film controls: PCDTPT (red), and PCDTPT* (blue).

Thin films spun-cast from 8 mg mL^–1^ solutions of PCDTPT* and PCDTPT in CB onto clean glass substrates exhibit absorption characteristics similar to each other and both are red shifted relative to what is observed in solution (Fig. 13). Thermal annealing does not significantly change the absorption profile (Fig. S10†). Surprisingly, CD spectroscopy shows no measurable signal for either PCDTPT or PCDTPT* films (Fig. 14). The absence of a CD signal indicates the failure of PCDTPT* to achieve chiral organization in the thin film. AFM topography images (Fig. S16†) show little difference between PCDTPT and PCDTPT* films, further emphasizing the absence of obvious morphological differences.

### Comparison of PCPDTBT* and PCDTPT*

Both PCPDTBT* and PCDTPT* form aggregated domains with chiral response in poor solvents. However, PCDTPT* assembles with a weaker degree of chiral order. Specifically, PCPDTBT* provides an average *g*_abs_ value one order of magnitude greater than PCDTPT* in solution within the absorption range attributed to aggregated chains. Even more revealing, PCPDTBT* exhibits a chiral response in the thin film whereas PCDTPT* does not. This suggests that the substitution of one –CH group for a –N group on the benzothiadiazole unit results in substantially different secondary structures for PCPDTBT* and PCDTPT*.

**Fig. 15:**
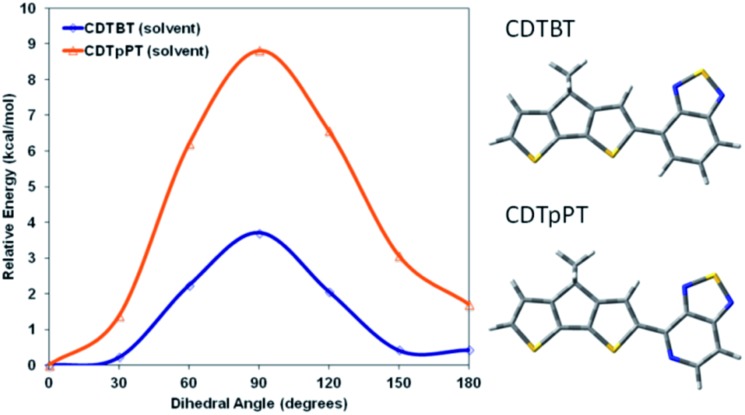
Calculated rotational barriers for CDT–BT (blue) and CDT–pPT (orange) in chlorobenzene.

It seemed reasonable to explore whether the higher degree of chiral order in PCPDTBT* compared to PCDTPT* may result from differences in the ability to form helical conformations and therefore on the rotational barriers between structural units in the backbone. A polymer coil with more conformational freedom would reasonably more readily achieve twisted conformations required in the helical secondary structure. Therefore, density functional theory (DFT), using the CAM-B3LYP functional and the 6-31G basis set was employed to estimate the rotational barrier of CDT–BT and CDT–pPT (pyridyl nitrogen) monomer units, see Fig. 15 for molecular substructures.^32^ The results using a dielectric function similar to chlorobenzene are provided in Fig. 15, while results calculated in vacuum are given in Fig. S18.† The calculated rotational barrier of CDT–pPT (∼9 kcal mol^–1^) is greater than CDT–BT (∼4 kcal mol^–1^). By extension, the rotational barriers of structural units in PCDTPT* are likely higher than those in PCPDTBT* based on the presence of the CDT–pPT structural units in the conjugated backbone.^33^,^34^ As a result, the lower rotational barrier of PCPDTBT*, relative to the stiffer PCDTPT*, would lower the energy requirements to acquire a helical conformation, providing a greater *g*_abs_ value.

## Conclusions

In conclusion, we provide the synthesis of two narrow bandgap conjugated polymers containing chiral 2-ethylhexyl side chains and backbone structures that have found widespread utility in the fabrication of organic (opto)electronic devices. The absorption and CD spectra of PCPDTBT* and PCDTPT* and comparison *versus* their non-chiral counterparts are consistent with chiral aggregates in solution. That one can achieve a similar fraction of aggregated chains for PCPDTBT* and obtain different *g*_abs_ values (Fig. 5) indicates that there is complexity in the internal structure of the aggregates. It appears that the degree of aggregation is temperature dependent and thermodynamically determined, whereas the fraction of more ordered chiral, and most likely helical, domains reflect the kinetics of the assembly process. As determined by GIWAXS examination, in the case of PCPDTBT*, the more homogenous molecular structure relative to PCPDTBT translates to a more ordered thin film upon solution deposition. The greater degree of order in the thin films of PCPDTBT* compared to PCPDTBT provides potential for higher charge-carrier mobilities. Thus future work to determine the performance of these materials in OFETs and OPV devices is warranted.

Despite their close structural similarities at the molecular level, PCDTPT* shows a smaller tendency to form chiral aggregates based on its smaller solution *g*_abs_ compared to PCPDTBT* and on the fact that it fails to provide a chiral response in the thin film. This difference may be rationalized if one assumes helical structures and by the calculated higher barrier to rotation within the CDT–pPT unit, compared to the CDT–BT unit. The higher rotational barrier contributes to a planar structure of PCDTPT*. The tendency toward a more planar structure for PCDTPT* is worth highlighting, since it may contribute to the polymer's high carrier mobility previously observed in aligned fiber OFETs, where charge transport occurs predominantly along the backbone direction. One should also consider whether helical backbone arrangements may be present in solid-state organizations of other common narrow bandgap conjugated polymers with racemic side chains. If so, our understanding of long range packing preferences that determine electronic coupling and the vast amount of detailed structural characterization of narrow bandgap conjugated polymers in the literature would need to be further refined.

## Supplementary Material

Supplementary information
