# Supplementary material for: Effect of chiral 2-ethylhexyl side chains on chiroptical properties of the narrow bandgap conjugated polymers PCPDTBT and PCDTPT
Source: Chem Sci. 2016 May 3;7(8):5313–21. doi: 10.1039/c6sc00908e (PMC6020614; doi:10.1039/c6sc00908e)
Supplement: Supplementary file 1 [file SC-007-C6SC00908E-s001.pdf]

Electronic Supplementary Material (ESI) for Chemical Science.
This journal is © The Royal Society of Chemistry 2016

Supporting Information for Effect of Chiral Ethylhexyl Side Chain on Chiroptical Properties of PCPDTBT and PCDTPT

S. L. Fronk, M. Wang, M. Ford, J. Coughlin, C.-K. Mai, and G. C. Bazan*

Materials and Methods

All reagents and solvents were purchased from Sigma Aldrich, BDM, Fisher Scientific, Alfa Aesar, or Acros Organics and used without further purification unless otherwise specified. Tetrakis(triphenylphosphine)palladium(0) [$Pd(PPh_3)_4$] was purchased from Strem Chemicals Inc. 4,7-dibromo-[1,2,5]thiadiazolo[3,4-c]pyridine (Br_2-PT) and 4,7-dibromo-[2,1,3]-benzothiadiazole (Br_2-BT) were purchased from Lumtec Co. *4H*-cyclopenta[2,1-*b*:3,4-*b'*]dithiophene (CDT) was purchased from WuXi AppTec Corporation. Deuterated solvents were purchased from Cambridge Isotopes Laboratories, Inc. Solvents used for air and/or water sensitive reactions (THF, toluene) were dried by passage through two columns of alumina and degassed by argon purge in a custom-built solvent purification system. Xylene at the ultra-dry grade was purchased from Acros Organics. Synthesis of EH-S-PCPDTBT and EH-S-PCDTPT were adapted from literature procedures.[1]

Microwave-assisted reactions were performed in a Biotage Initiator TM microwave reactor. ^{1}HNMR spectra were obtained on a Varian VNMRS 500 MHz or 600 MHz spectrometer at room temperature. GPC chromatographs were obtained using a Waters 2690 Separation Module with two Agilent PLGEL 5μm, MIXED-D columns running $CHCl_3$/0.25% triethylamine as eluent. Molecular weights were calculated relative to linear PS standards. MALDI spectra were obtained on a Bruker Microflex series MALDI-TOF using a matrix of dithranol saturated chloroform.

Synthesis of (S)-2-ethylhexan-1-ol

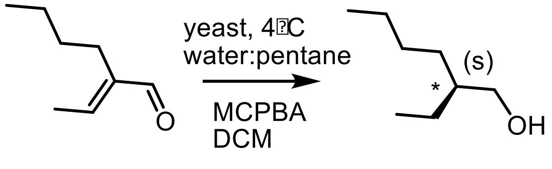

2-ethylidenehexanal **(S)-2-ethylhexan-1-ol**

Scheme S1. Synthesis of (S)-2-ethylhexan-1-ol.

(S)-2-ethylhexan-1-ol was synthesized according to literature procedures.[2] HK_2PO_4 (16.647 g, 95.6 mmol), H_2KPO_4 (0.720 g, 5.29 mmol), and sucrose (10.280 g, 30.0 mmol) were dissolved in Millipore water (1000 mL). Yeast (200. g) was added slowly while stirring. The yeast mixture was allowed to stir for 2.5 hours. Pentane (1500 mL) was added. 2-ethylidene hexanal (10.254 g, 81.3 mmol) was added. The reaction was allowed to stir at 4°C for 1 week. The aqueous/yeast layer was removed by centrifugation. The resulting organic layer was filter through celite, concentrated under reduced pressure, and dried under vacuum. The resulting oil was dissolved in DCM (100 mL) and cooled to 0°C. MCPBA (7.904 g, 45.8 mmol) was added. The reaction was stirred for two hours. The reaction mixture was washed with water, dried over sodium sulfate, and concentrated under reduced pressure. The crude product was first purified by distillation. The product was then purified by column chromatography using 6:4 DCM:pentane as the eluent (R_f ~ 0.25). (S)-2-ethylhexan-1-ol was produced in 21% yield (2.181 g). ^{1}H NMR (500 MHz, $CDCl_3$) δ (ppm): 3.53 (d, 2H), 1.41-1.20 (m, 10H), 0.88 (t, 6H).

^{13}C NMR (600 MHz, $CDCl_3$) δ (ppm): 65.31, 41.95, 30.12, 29.09, 23.32, 23.08, 14.09, 11.12. GC/MS: calculated for $C_8H_{18}O$ (m/z): 130.14, found 130. $[\alpha]_D$ = +2.954° (Concentration of 1.2 g/100 ml in acetone, 25 °C). ([Ref][3]: $[\alpha]_D$ = +3.1°, e.e. = 95%).

Chiral Derivatization of (S)-2-ethylhexan-1-ol

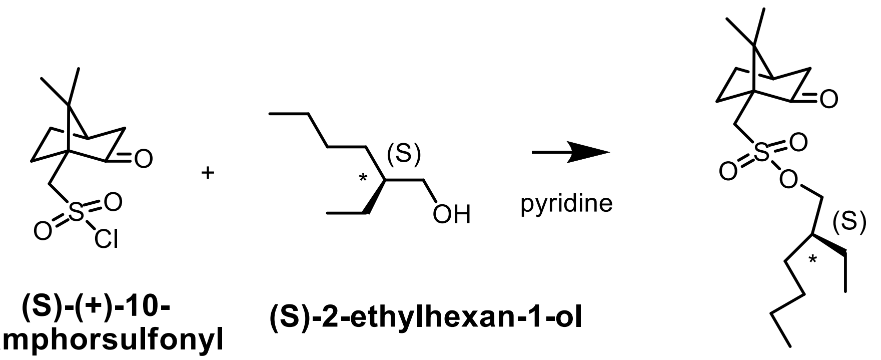

(S)-(+)-10-camphorsulfonyl chloride **(S)-2-ethylhexan-1-ol**

Scheme S2. Chiral derivatization reaction of (S)-(+)-10-camphorsulfonyl chloride and (S)-2-ethylhexan-1-ol.

(S)-2-ethylhexan-1-ol (0.0554 g, 0.425 mmol) and (S)-(+)-10-camphorsulfonyl chloride (0.1452 g, 0.579 mmol) were dissolved in pyridine (1 mL). The reaction was covered in aluminum foil and allowed to stir for about 16 hours. Pyridine was removed under reduced pressure. The mixture was separated using column chromatography with hexane:ethyl acetate (1:1) as the eluent (R_f ~ 0.4). The chiral adduct was produced at 79% yield (0.116 g). ^1H NMR (500 MHz, $CDCl_3$) δ (ppm): 4.16 (d, 2H), 3.57 (d, 1H), 2.96 (d, 1H), 2.48 (m, 1H), 2.36 (m, 1H), 2.10 (t, 1H), 2.01 (m, 1H), 1.92 (d, 1H), 1.66-1.57 (m, 2H), 1.46-1.34 (m, 3H), 1.33-1.19 (m, 6H), 1.10 (s, 3H), 0.87 (m, 9H).

Chiral Derivatization of 2-ethylhexan-1-ol

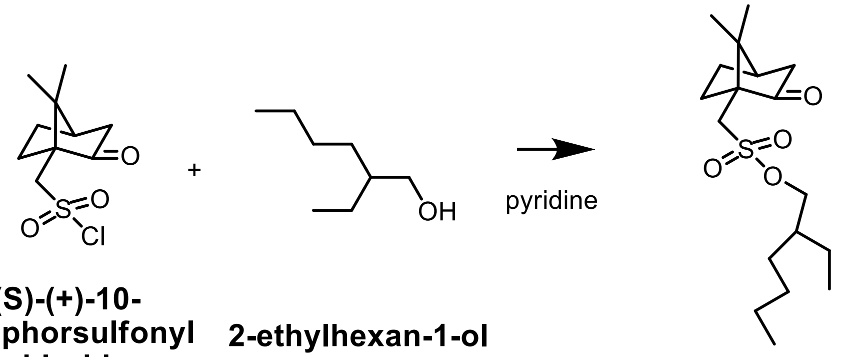

(S)-(+)-10-camphorsulfonyl chloride **2-ethylhexan-1-ol**

Scheme S3. Chiral derivatization reaction of (S)-(+)-10-camphorsulfonyl chloride and 2-ethylhexan-1-ol.

2-ethylhexan-1-ol (0.1149 g, 0.882 mmol) and (S)-(+)-10-camphorsulfonyl chloride (0.2895 g, 1.15 mmol) were dissolved in pyridine (2 mL). The reaction was covered in aluminum foil and allowed to stir for about 16 hours. Pyridine was removed under reduced pressure. The mixture was purified using column chromatography with hexane:ethyl acetate (8:2) as the eluent (R_f ~ 0.4). The racemic adduct was produced at 82% yield (0.249 g). ^1H NMR (500 MHz, $CDCl_3$) δ (ppm): 4.13 (m, 2H), 3.54 (d, 1H), 2.93 (d,

1H), 2.44 (m, 1H), 2.33 (m, 1H), 2.07 (t, 1H), 2.00 (m, 1H), 1.90 (d, 1H), 1.64-1.53 (m, 2H), 1.43-1.31 (m, 3H), 1.30-1.16 (m, 6H), 1.07 (s, 3H), 0.84 (m, 9H).

Synthesis of (S)-3-(bromomethyl)heptane

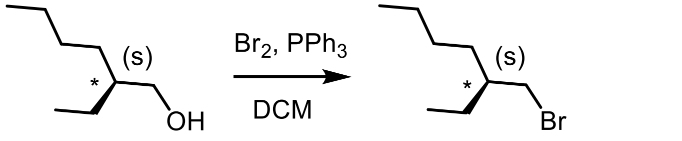

(S)-2-ethylhexan-1-ol **(S)-3-(bromomethyl)heptane**

Scheme S4. Synthesis of (S)-3-(bromomethyl)heptane.

PPh_3 (2.7407 g, 10.4 mmol) was added to a 100 mL round bottom flask and dissolved in DCM (21 mL). The solution was cooled to 0°C. Elemental Br_2 (3.5408 g, 22.2 mmol) was added dropwise to the stirring solution. (S)-2-ethylhexan-1-ol (1.0559 g, 8.11 mmol) was dissolved in pyridine (1.54 mL) and added dropwise to the stirring reaction mixture. The reaction was allowed to stir for 1 hour at 0°C. The ice bath was then removed and the reaction was allowed to stir at room temperature for 1 hour. Na_2SO_3 (10% aq solution) was added to quench the reaction. The reaction mixture was washed with water and the organic layer was dried over sodium sulfate. The solvent was removed under reduced pressure. The resulting mixture was separated using column chromatography with hexane:DCM (8:2) as the eluent (R_f ~ 0.8). (S)-3-(bromomethyl)heptane was produced as a colorless oil with 69% yield (1.02 g). ^1H NMR (500 MHz, $CDCl_3$) δ (ppm): 3.46 (m, 2H), 1.54 (m, 1H), 1.46-1.22 (m, 8H), 0.90 (t, 6H). ^{13}C NMR (600 MHz, $CDCl_3$) δ (ppm): 41.06, 39.12, 31.86, 28.82, 25.14, 22.86, 14.06, 10.86. GC/MS: calculated for $C_8H_{17}Br$ (m/z): 192.05, found 192.

Synthesis of EH-S-CDT

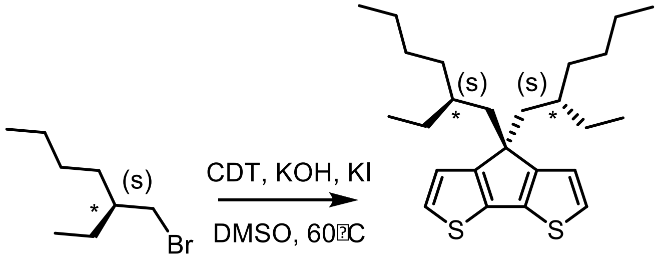

(S)-3-(bromomethyl)heptane **EH-S-CDT**

Scheme S5. Synthesis of EH-S-CDT.

Cyclopentadithiophene (0.4373 g, 2.45 mmol), potassium iodide (87.9 mg, cat.), and potassium hydroxide powder (0.499 g, 8.89 mmol) were dissolved in DMSO (10 mL). The solution was degassed with argon for 15 minutes. (S)-3-(bromomethyl)heptane (1.069 g, 5.54 mmol) was added via syringe. The reaction mixture was heated to 60°C and allowed to stir for about 16 hours. The reaction was cooled to room temperature and diluted with hexane. The reaction mixture was poured over DI water, washed with water, and dried over sodium sulfate. The solvent was removed under reduced pressure. The resulting red-brown oil was purified using column chromatography with hexane as the eluent (R_f ~ 0.8). EH-S-CDT was isolated as a colorless oil and produced at 88% yield. ^1H NMR (500 MHz, $CDCl_3$) δ (ppm): 7.09 (d, 2H), 6.91 (d, 2H), 1.86 (m, 4H), 1.05-0.82 (m, 16H), 0.74 (t, 4H), 0.58 (t, 6H). ^{13}C NMR

(600 MHz, $CDCl_3$) δ (ppm): 157.65, 136.78, 123.95, 122.28, 53.26, 43.26, 34.98, 34.19, 28.55, 27.27, 22.73, 14.04, 10.65. GC/MS: calculated for $C_{25}H_{38}S_2$ (m/z): 402.25, found 402.

Stannylation of EH-S-CDT

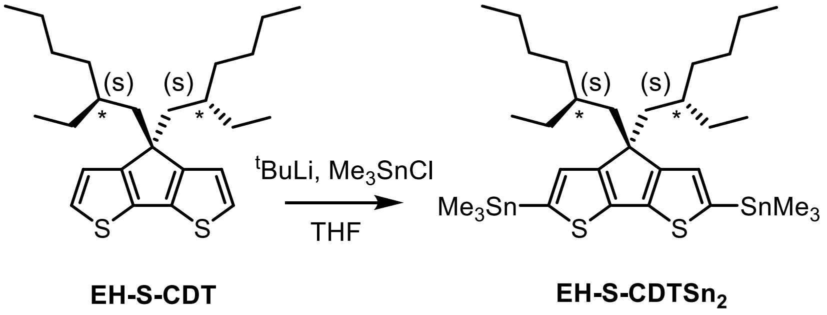

Scheme S6. Stannylation of EH-S-CDT.

In a nitrogen-filled glovebox, EH-S-CDT (0.1542 g, 0.383 mmol) was dissolved in THF (5 mL). ^{t}BuLi (0.90 mL, 1.7M in pentane) was added slowly and the reaction was allowed to stir for 1 hour. Me_3SnCl (0.4042 g, 2.03 mmol) was dissolved in THF (5 mL). The Me_3SnCl solution was slowly added and the reaction was allowed to stir for about 16 hours. The reaction was removed from the glovebox and water (5 mL) was added to quench. The reaction mixture was diluted with hexane, washed with water, and dried over sodium sulfate. The solvent was removed under reduced pressure to give a colorless oil. EH-S-CDTSn$_2$ was produced with an 82% yield (0.221 g). ^{1}H NMR (500 MHz, $CDCl_3$) δ (ppm): 7.00 (s, 2H), 1.87 (m, 4H), 1.03-0.84 (m, 18H), 0.75 (t, 6H), 0.60 (t, 6H), 0.37 (s, 18H). ^{13}C NMR (600 MHz, $CDCl_3$) δ (ppm): 159.66, 142.62, 136.17, 130.15, 104.98, 52.12, 43.18, 35.15, 34.45, 28.68, 27.59, 22.89, 16.90, 14.16, 10.73, -8.20.

Polymerization of BT-Br$_2$ and EH-S-CDTSn$_2$

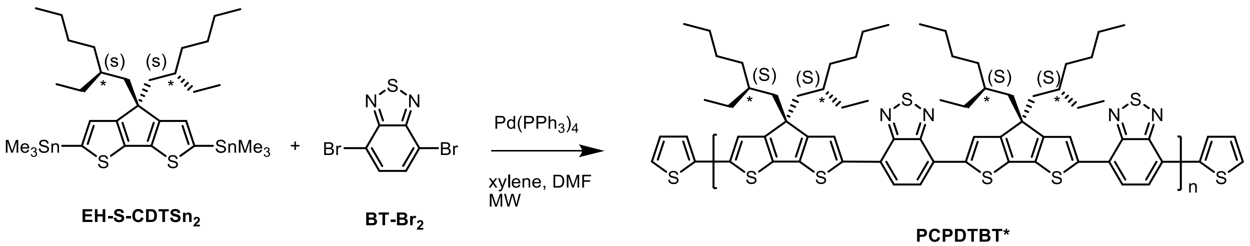

Scheme S7. Stille-coupling polymerization of EH-S-CDTSn$_2$ and BT-Br$_2$.

In a nitrogen-filled glovebox, EH-S-CDTSn$_2$ (0.055 g, 0.098 mmol), BT-Br$_2$ (0.0302 g, 0.103 mmol), and $Pd(PPh_3)_4$ (0.0113 g, 0.0098 mmol) were dissolved in dry xylenes (3 mL) in a microwave tube. DMF (0.2 mL) was added. The microwave tube was sealed and removed from the nitrogen glovebox. Microwave-assisted Stille-coupling was performed using the following procedure: 80°C for 5 minutes, 120°C for 5 minutes, 140°C for 5 minutes, and 170°C for 40 minutes. The reaction was allowed to cool to room temperature and returned to nitrogen atmosphere. 2-bromothiophene (0.15 mL, 0.155 mmol) and $Pd(PPh_3)_4$ (0.0043 g, 0.0037 mmol) were dissolved in xylene (1.5 mL) and added to the microwave tube. The reaction was allowed to stir at 150°C in an oil bath for about 16 hours. After cooling to room temperature, the polymer, PCPDTBT* was precipitated with methanol, filter, and dried under vacuum. The polymer was purified by Soxhlet extraction by washing successively with methanol (2 hours),

acetone (6 hours), hexane (16 hours) and collected in chloroform (9 hours). The solvent was removed under reduced pressure to produce a black solid with 46% yield (0.024 g). ^{1}H NMR (500 MHz, $CDCl_3$) δ (ppm): 8.16 (s, 2H), 7.91 (s, 2H), 2.10 (s, 2H), 1.08 (m, 20H), 0.74 (m, 12H). GPC ($CHCl_3$): M_n = 23,132; M_w = 36,632; PDI = 1.6.

Stille-coupling of PT-Br_2 and EH-S-CDTSn$_2$

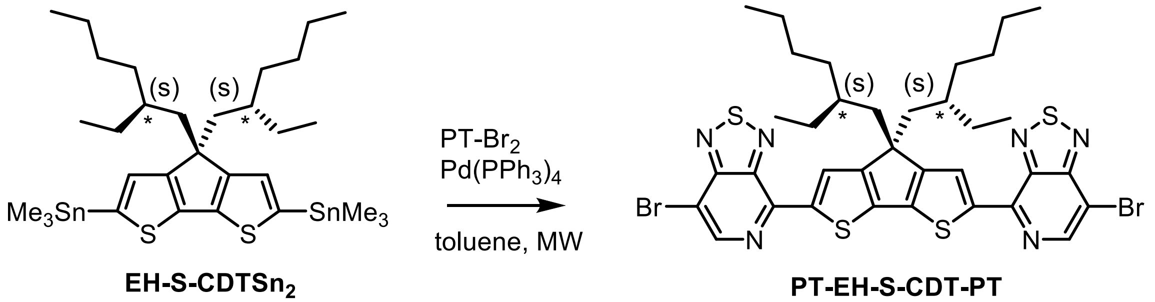

Scheme S8. Stille-coupling of PT-Br_2 and EH-S-CDTSn$_2$.

In a nitrogen-filled glovebox, EH-S-CDTSn$_2$ (0.3161 g, 0.434 mmol), PT-Br_2 (0.2625 g, 0.890 mmol), and $Pd(PPh_3)_4$ (0.050 g, 0.0433 mmol) were dissolved in dry toluene (11 mL) in a microwave tube. The microwave tube was sealed and removed from the nitrogen glovebox. Microwave-assisted Stille coupling was performed using the following procedure: 120°C for 10 minutes, 140°C for 10 minutes, 160°C for 10 minutes, and 170°C for 40 minutes. The reaction was cooled to room temperature and diluted with $CHCl_3$. The reaction mixture was extracted with $CHCl_3$, washed with water, and dried over sodium sulfate. The solvent was removed under reduced pressure. The resulting mixture was purified using column chromatography using a solvent gradient beginning with $CHCl_3$:hexane (1:1) and progressing to $CHCl_3$ (R_f ~ 0.3). The resulting purple solid was isolate with 67% yield (0.228 g). NMR and mass spec data. ^{1}H NMR (500 MHz, $CDCl_3$) δ (ppm): 8.68 (s, 2H), 8.62 (s, 2H), 2.11 (m, 4H), 1.08-0.91 (m, 16H), 0.83 (m, 2H), 0.66-0.57 (m, 12H). ^{13}C NMR (600 MHz, $CDCl_3$) δ (ppm): 161.76, 156.38, 147.88, 147.68, 146.00, 146.00, 143.20, 127.74, 107.44, 54.49, 43.13, 35.47, 34.29, 28.56, 27.59, 22.83, 14.06, 10.77. MALDI: calculated for $C_{35}H_{38}Br_2N_6S_4$ (m/z): 828.041, found 831.

Polymerization of PT-EH-S-CDT-PT and EH-S-CDTSn$_2$

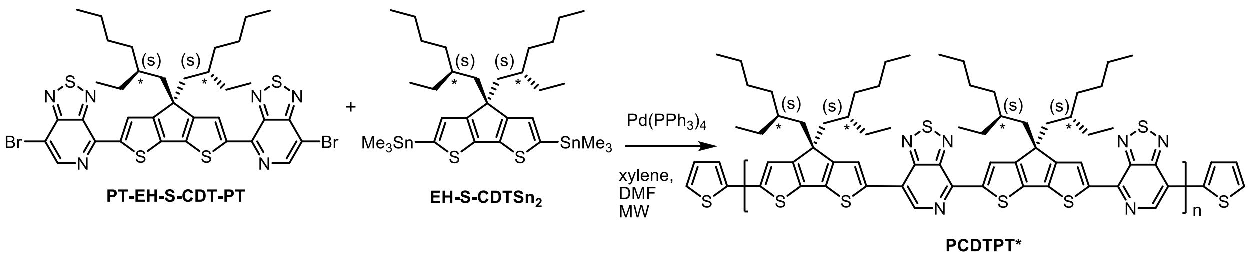

Scheme S9. Stille-coupling polymerization of PT-EH-S-CDT-PT and EH-S-CDTSn$_2$.

In a nitrogen-filled glovebox, EH-S-CDTSn$_2$ (0.0702 g, 0.0964 mmol), PT-EH-S-CDT-PT (0.075 g, 0.0903 mmol), and $Pd(PPh_3)_4$ (0.0063 g, 0.00545 mmol) were dissolved in dry xylenes (3.5 mL) in a microwave tube. DMF (0.2 mL) was added. The microwave tube was sealed and removed from the nitrogen glovebox. Microwave-assisted Stille coupling was performed using the following procedure: 80°C for 5 minutes, 130°C for 5 minutes, 170°C for 5 minutes, and 200°C for 40 minutes. The reaction was cooled

to room temperature and returned to nitrogen atmosphere. 2-bromothiophene (0.15 mL, 1.55 mmol) and $Pd(PPh_3)_4$ (0.0040 g, 0.00346 mmol) were dissolved in xylenes (1.5 mL) and added to the microwave tube. The reaction was allowed to stir at 150°C in an oil bath for about 16 hours. After cooling to room temperature, the resulting polymer, PCDTPT* was precipitated with methanol, filtered, and dried under vacuum. The polymer was purified by Soxhlet extraction by washing successively with methanol (2 hours), acetone (6 hours), hexane (16 hours), and collected in chloroform (8 hours). The solvent was removed under reduced pressure to form a purple solid with 85% yield (0.082 g). ^{1}H NMR (600 MHz, $CDCl_3$) δ (ppm): 8.86 (s, 2H), 8.65 (s, 2H), 8.10 (s, 2H), 2.22-0.61 (m, 132H). GPC ($CHCl_3$): M_n = 17,415; M_w = 30,194; PDI = 1.7.

Synthesis of PCPDTBT

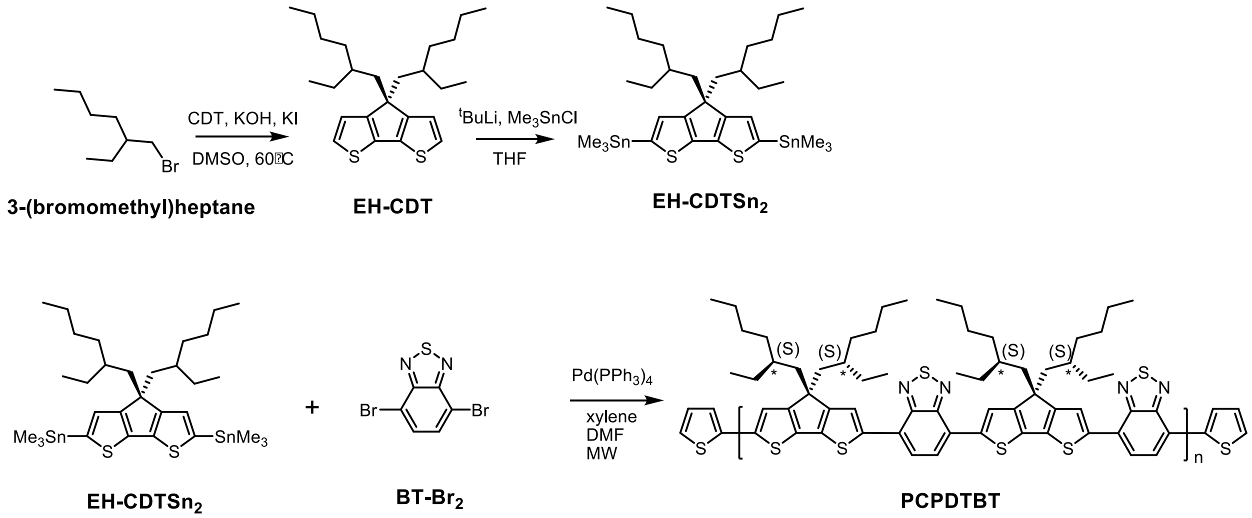

Scheme S10. Synthesis of PCPDTBT.

The procedure for synthesizing PCPDTBT is the same as that of PCPDTBT* where (S)-3-(bromomethyl)heptane is replaced by 3-(bromomethyl)heptane. GPC ($CHCl_3$): M_n = 31,553; M_w = 71,410; PDI = 2.3.

Synthesis of PCDTPT

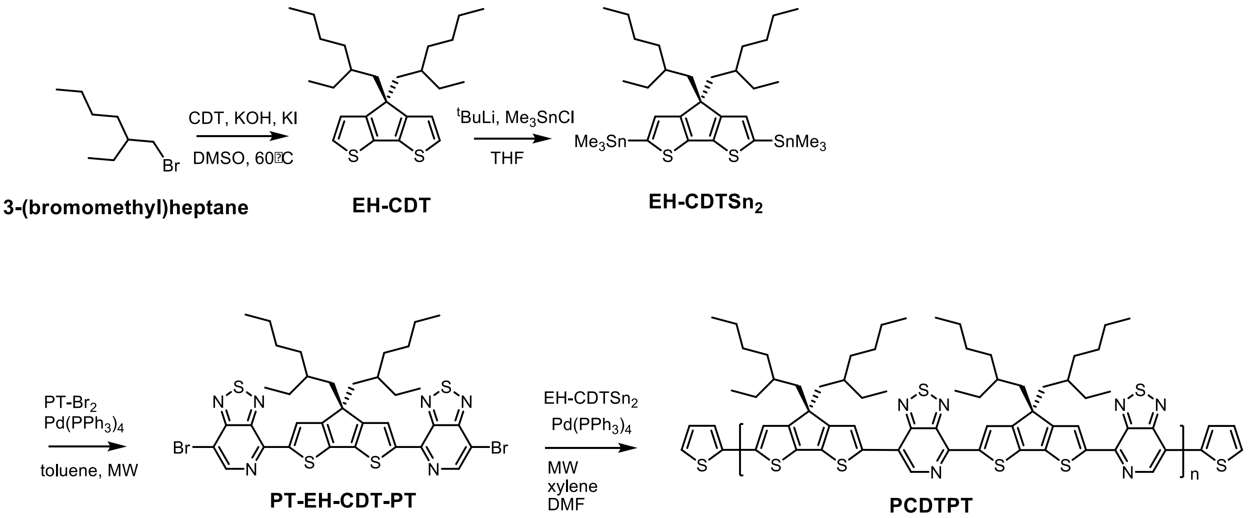

Scheme S11. Synthesis of PCDTPT.

The procedure for synthesizing EH-PCDTPT is the same as that of EH-S-PCDTPT where (S)-3-(bromomethyl)heptane is replaced by 3-(bromomethyl)heptane. GPC ($CHCl_3$): M_n = 22,506; M_w = 44,405; PDI = 2.0.

^{1}H NMR spectra of chiral derivatization reactions

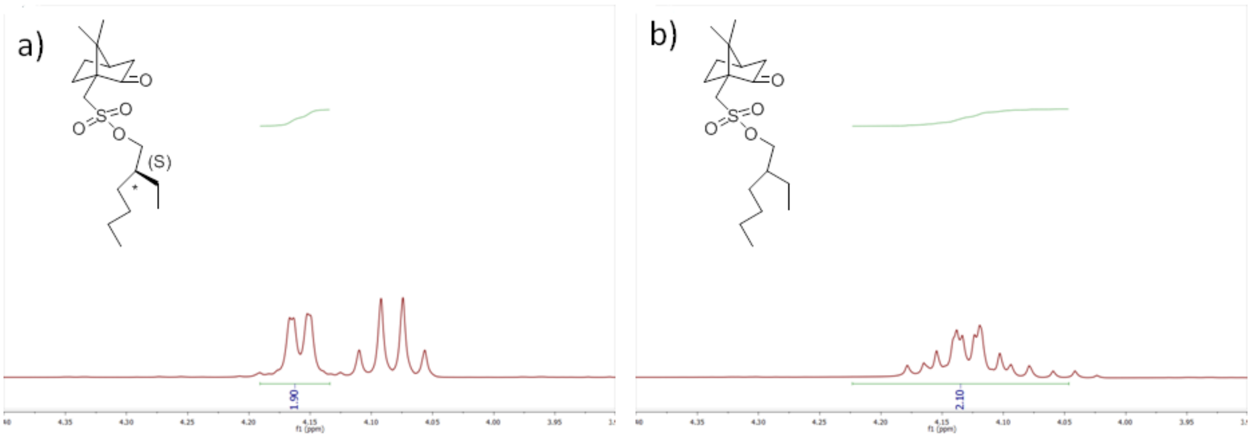

Figure S1. ^{1}HNMR spectra of a) chiral adduct and b) racemic adduct.

Differential Scanning Calorimetry

Differential scanning calorimetry was measured a TA Instruments DSC (Model Q-20) with about 5 mg of polymer sample at a rate of 10°C/minute in the temperature range of 20°C to 300°C.

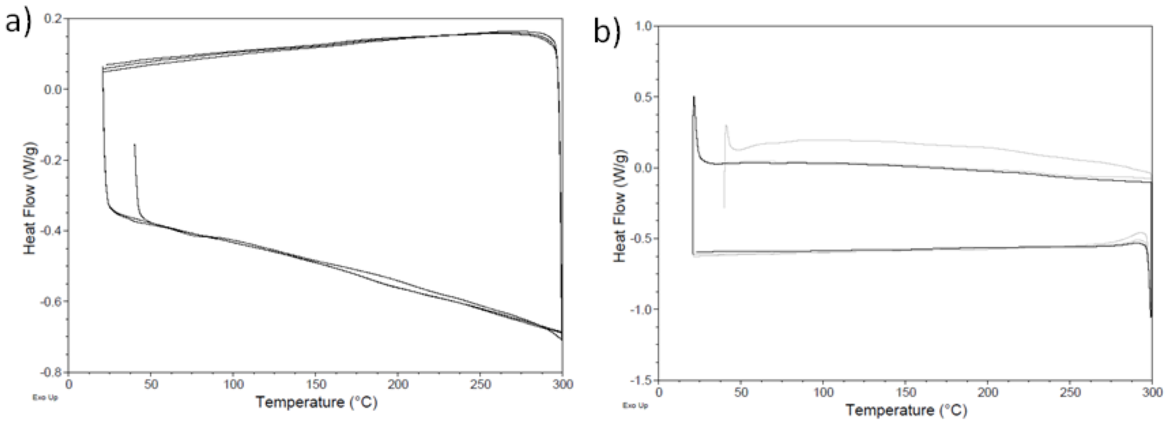

Figure S2. Differential scanning calorimetry curves of a) PCPDTBT and b) PCPDTBT*.

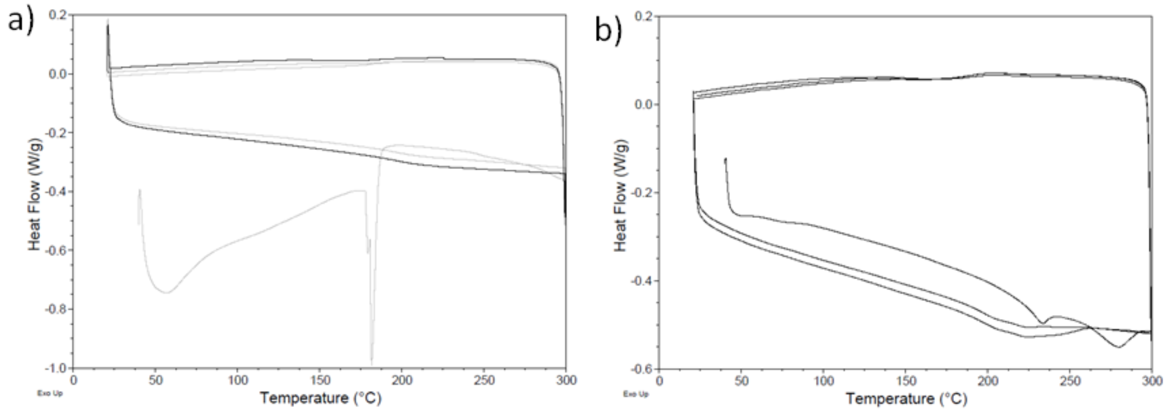

Figure S3. Differential scanning calorimetry curves of a) PCDTPT and b) PCDTPT*.

Optical Absorption

Optical absorption spectra were measured using a Perkins Elmer Lambda 750 UV-Vis Spectrometer containing a STD detection module. Solvents for solution spectra were used without further purification. Solutions were measured using 10 mm path length quartz cuvettes. Solutions were prepared in CB, 1:1 CB:DIO, or 8:2 CB:DMSO at concentrations varying from 0.01 mg/mL to 0.07 mg/mL. Optical absorption measurements were taken at room temperature unless otherwise indicated. Thin films were fabricated using spin-coating in a nitrogen-filled glovebox at room temperature by depositing 20 µL of 8 mg/mL CB solution on a clean glass substrate and spinning for 120 seconds at 750 rpm.

Temperature-dependent optical absorption spectroscopy: Solutions were heating using a PolyScience Digital Temperature Controller. Solutions were heated to the desired temperature, allowed to equilibrate for 5 minutes, and then the optical absorption spectrum was measured.

The oscillator strength was calculated using the equation f = $(4.3X10^{-9})*\int\varepsilon dv$ where $\int\varepsilon dv$ is the integrated extinction coefficient with v in wave numbers. $\int\varepsilon dv$ was calculated by fitting the peaks with a Gaussian function to de-convolute the peaks and determine the area under the curve.

Table S1: Oscillator Strength of PCPDTBT and PCPDTBT*

Material	Solvent	$\pi\rightarrow\pi^*$	0-0
PCPDTBT	1:1 CB:DIO	13.2	11.7
PCPDTBT*	CB	26.6	9.4
PCPDTBT*	1:1 CB:DIO	7.3	1.7

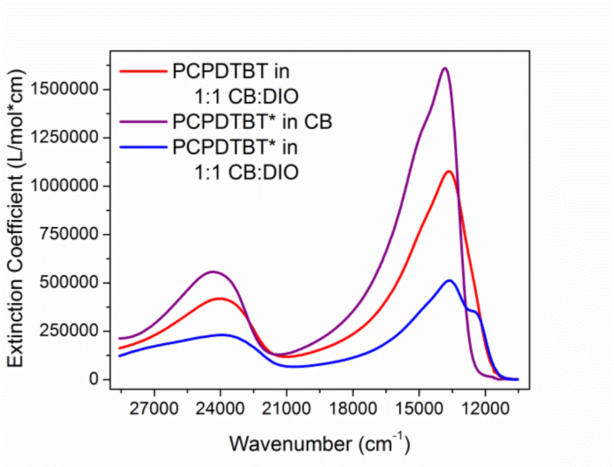

Figure S4: Extinction coefficients as a function of wave number for solutions of PCPDTBT in 1:1 CB:DIO (red) and PCPDTBT* in CB (purple) and 1:1 CB:DIO (blue).

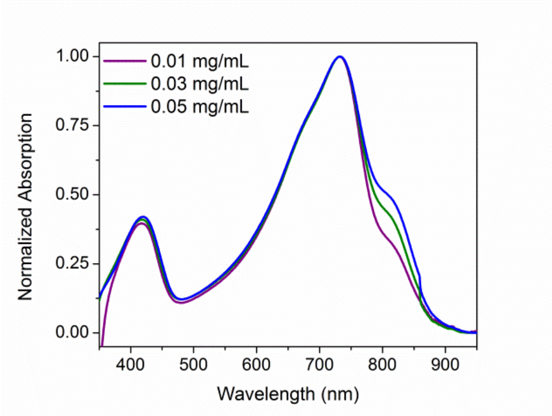

Figure S5. Concentration-dependent normalized absorption spectra of PCPDTBT* solutions at 0.01 mg/mL (purple), 0.03 mg/mL (green), and 0.05 mg/mL (blue) in 1:1 CB:DIO.

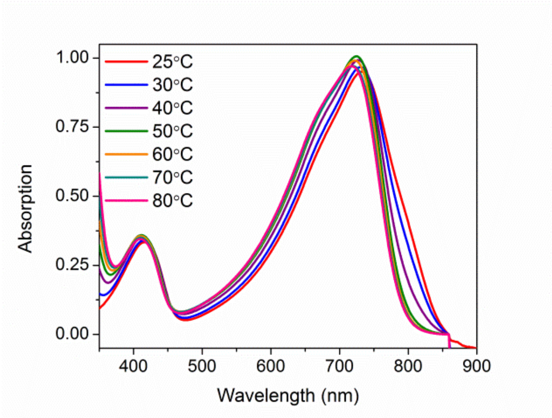

Figure S6. Temperature-dependent absorption of PCPDTBT at 0.03 mg/mL in 1:1 CB:DIO.

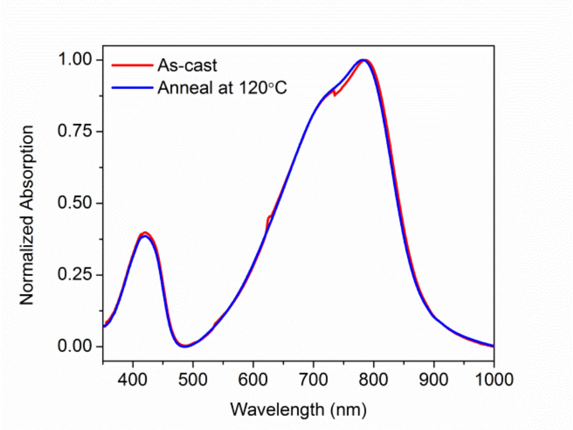

Figure S7. Normalized thin film absorption spectra of PCPDTBT* before (red) and after (blue) annealing at 120°C for 10 minutes.

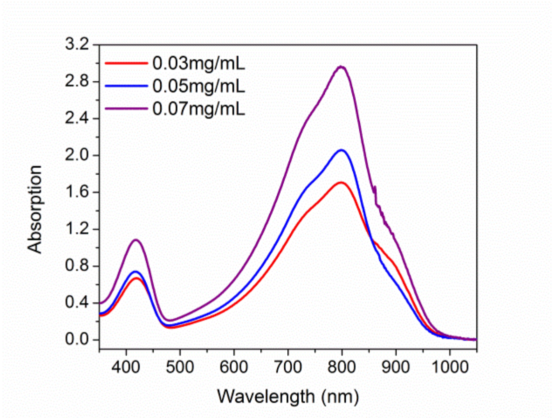

Figure S8. Concentration-dependent absorption of PCDTPT* at 0.03 mg/mL (red), 0.05 mg/mL (blue), and 0.07 mg/mL (purple) in 8:2 CB:DMSO.

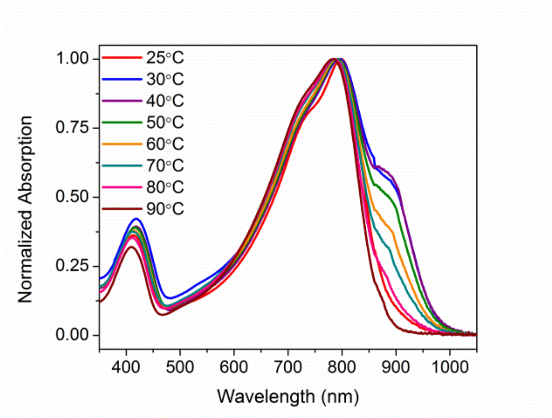

Figure S9. Temperature-dependent normalized absorption of PCDTPT at 0.01 mg/mL in 8:2 CB:DMSO.

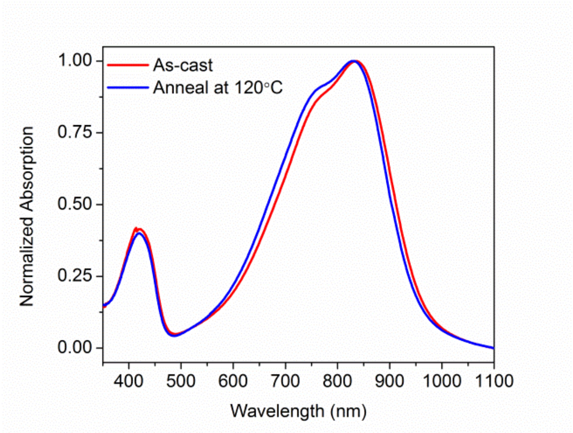

Figure S10. Normalized thin film absorption spectra of PCDTPT* before (red) and after (blue) annealing at 120°C for 10 minutes.

CD Spectroscopy

Circular dichroism spectra were measured using an Aviv Circular Dichroism Spectrometer, Model 202, which contains a Xenon lamp. Circularly polarized light is produced by a 50 kHz photoelastic modulator. Temperature is controlled via Peltier thermoelectric heating and cooling system. Solvents for solution spectra were used without further purification. Solutions were measured using 10 mm path length quartz cuvettes. Solutions were prepared in CB, 1:1 CB:DIO, or 8:2 CB:DMSO at concentrations varying from 0.01 mg/mL to 0.07 mg/mL. Thin films were fabricated using spin-coating in a nitrogen-filled glovebox at room temperature by depositing 20 µL of 8 mg/mL CB solution on a clean glass substrate and spinning for 120 seconds at 750 rpm.

Cooling kinetics: An initial CD spectrum was recorded at room temperature. The temperature was then increased at 5°C/min from 25°C to 90°C. The solution was held at 90°C for 15 minutes. The solution was cooled at a controlled rate of 1°C/min or 5°C/min from 90°C back down to 25°C. As soon as cooling began, the CD signal at 870 nm was monitored. CD signal was again measured one week after the cooling experiments.

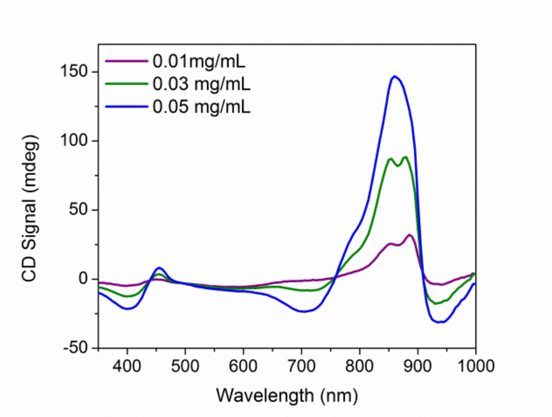

Figure S11. Concentration-dependent CD spectra of PCPDTBT* at 0.01 mg/mL (purple), 0.03 mg/mL (green), and 0.05 mg/mL (blue) in 1:1 CB:DIO.

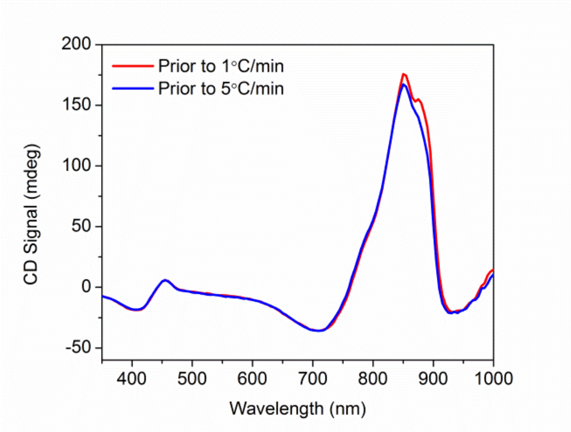

Figure S12. CD spectra of PCDTBT* prior to cooling at 1°C/minute (red) and 5°C/minute (blue).

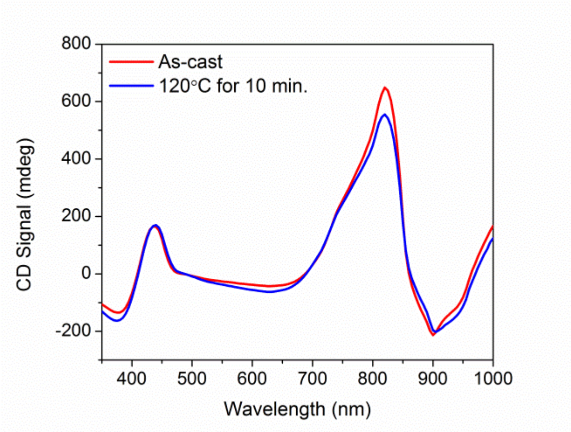

Figure S13. CD spectra of PCPDTBT* thin films before (red) and after (blue) annealing at 120°C for 10 minutes.

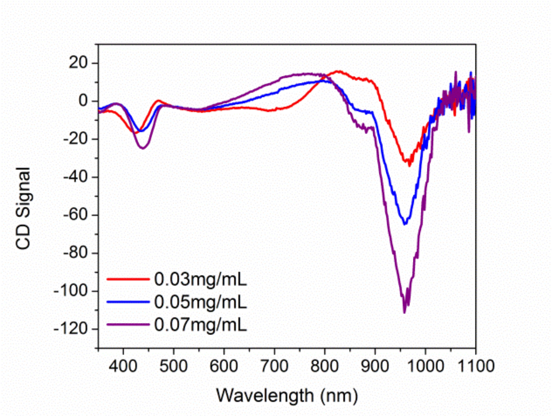

Figure S14. Concentration-dependent CD spectra of PCDTPT* at 0.03 mg/mL (red), 0.05 mg/mL (blue), and 0.07 mg/mL (purple) in 8:2 CB:DMSO.

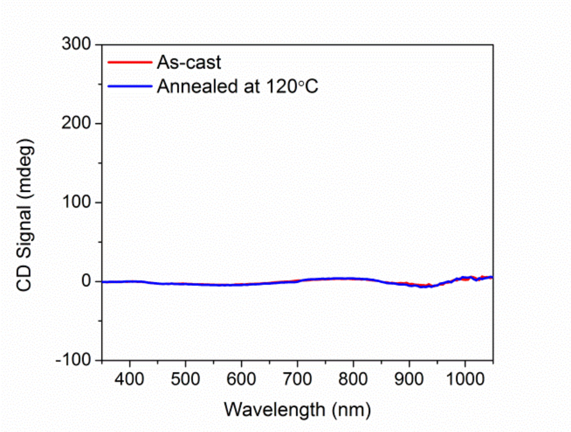

Figure S15. CD spectra of PCDTPT* thin films before (red) and after (blue) annealing at 120°C for 10 minutes.

AFM

Atomic force microscopy images were obtained using an Asylum MFP-3D AFM controlled through Igor Pro software. The AFM tips used were NanoWorld Pointprobe Al-coated, non-contact mode Si cantilevers, with a resonant frequency of 190 kHz and a spring constant of 48 N/m. All images were taken in air using AC mode. Thin films were fabricated using spin-coating in a nitrogen-filled glovebox at room temperature by depositing 20 μL of 8 mg/mL CB solution on a clean glass substrate and spinning for 120 seconds at 750 rpm.

Table S2: Average RMS Values for PCDTPT and PCDTPT* Films

Material	Avg. RMS (nm)	Error based on multiple measurements (nm)	Avg. error provided by AFM software (nm)
PCDTPT*	3.0	+/- 0.85	+/- 1.84
PCDTPT	1.4	+/- 0.55	+/- 0.81

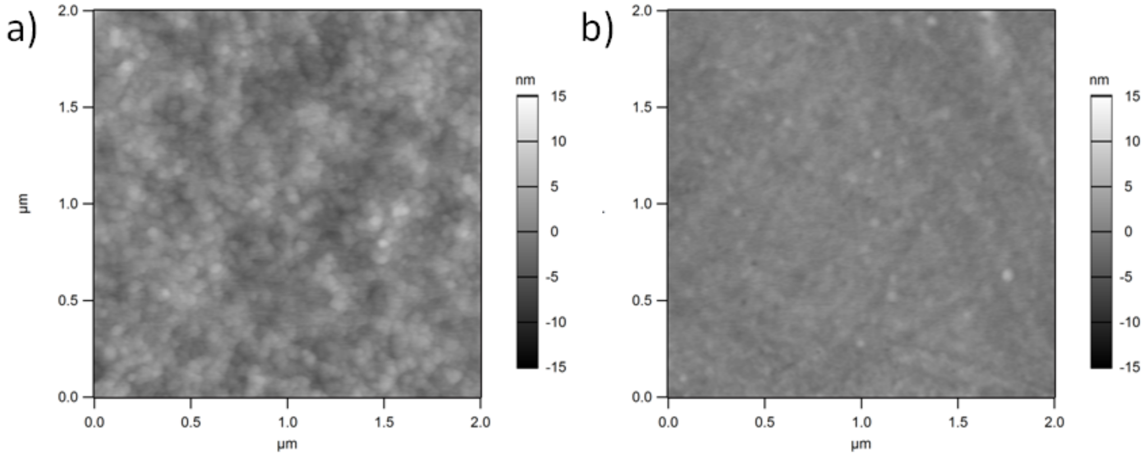

Figure S16. AFM topography images of a) PCDTPT* and b) PCDTPT.

GIWAXS

Samples were obtained as thin films by spin-coating 8 mg/mL CB solutions of PCDTBT* and PCDTBT onto silicon wafers. The one-dimensional and two-dimensional GIWAXS profiles were taken in the nominally in-plane and out-of-plane directions.

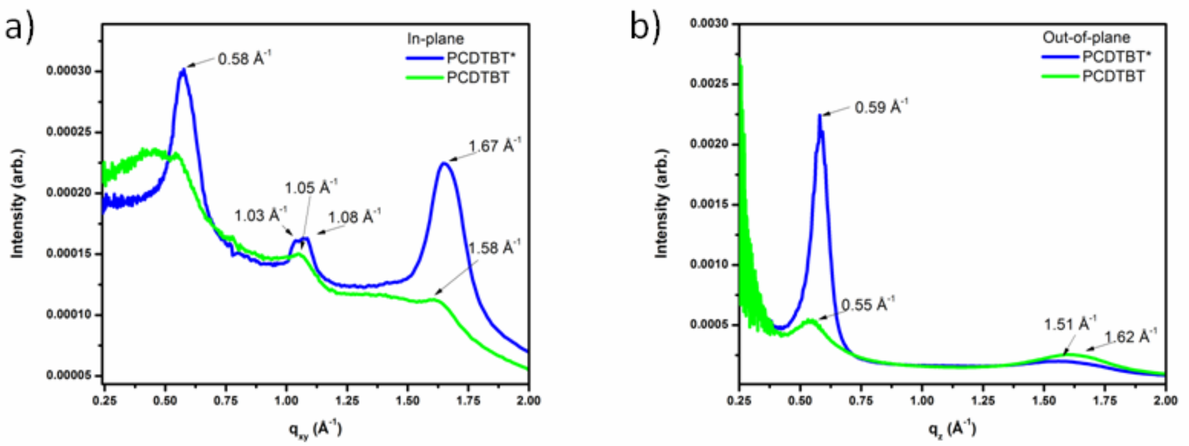

Figure S17. GIWAXS line cut profiles of PCDTBT* (blue) and PCDTBT (green) in the a) in-plane and b) out-of-plane directions. Relevant peaks have been labeled with their corresponding q values.

Density Functional Theory (DFT) Calculations

Calculations were done using the Gaussian 09 software suite.[4] The hybrid long range corrected CAM-B3LYP functional[5] with the basis set 6-31G(d,p) were used for the optimization of the geometry.

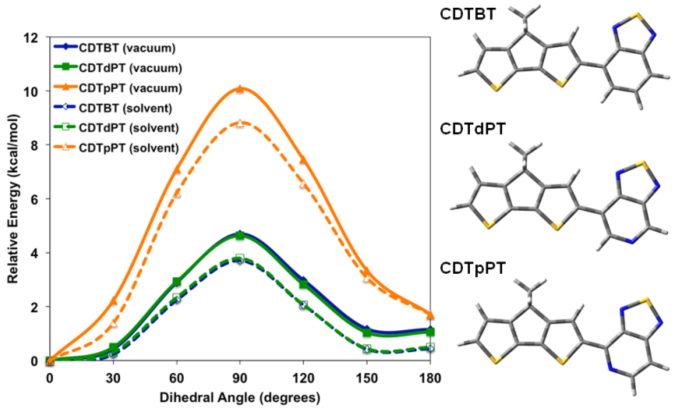

Figure S18. Calculated rotational barriers for CDTBT (blue), CDTdPT (green) and CDTpPT (orange) in vacuum (solid) and in chlorobenzene (dashed).

References

1. a) Coffin, R. C.; Peet, J.; Rogers, J.; Bazan, G. C. *Nature Chemistry*, **2009**, *1*, 657-661. b) Ying, L.; Hsu, B. B. Y.; Zhan, H.; Welch, G. C.; Zalar, P.; Perez, L. A.; Kramer, E. J.; Nguyen, T. Q.; Heeger, A. J.; Wong, W. Y.; Bazan, G. C. *J. Am. Chem. Soc.* **2011**, *133*, 18538-18541.
2. Huang, Y.; Zhang, F.; Gong, Y. *Tetrahedron Letters.* **2005**, *46*, 7217-7219.
3. Grenier, C. R.; George, S. J.; Joncheray, T. J.; Meijer, E. W.; Reynolds, J. R. *J. Am. Chem. Soc.* **2007**, *129*, 10694-10699.
4. Frisch, M. J.; Trucks, G. W.; Schlegel, H. B.; Scuseria, G. E.; Robb, M. A.; Cheeseman, J. R.; Scalmani, G.; Barone, V.; Mennucci, B.; Petersson, G. A.; Nakatsuji, H.; Caricato, M.; Li, X.; Hratchian, H. P.; Izmaylov, A. F.; Bloino, J.; Zheng, G.; Sonnenberg, J. L.; Hada, M.; Ehara, M.; Toyota, K.; Fukuda, R.; Hasegawa, J.; Ishida, M.; Nakajima, T.; Honda, Y.; Kitao, O.; Nakai, H.; Vreven, T.; Montgomery, J.; Peralta, J. E.; Ogliaro, F.; Bearpark, M.; Heyd, J. J.; Brothers, E.; Kudin, K. N.; Staroverov, V. N.; Kobayashi, R.; Normand, J.; Raghavachari, K.; Rendell, A.; Burant, J. C.; Iyengar, S. S.; Tomasi, J.; Cossi, M.; Rega, N.; Millam, J. M.; Klene, M.; Knox, J. E.; Cross, J. B.; Bakken, V.; Adamo, C.; Jaramillo, J.; Gomperts, R.; Stratmann, R. E.; Yazyev, O.; Austin, A. J.; Cammi, R.; Pomelli, C.; Ochterski, J. W.; Martin, R. L.; Morokuma, K.; Zakrzewski, V. G.; Voth, G. A.; Salvador, P.; Dannenberg, J. J.; Dapprich, S.; Daniels, A. D.; Farkas, Ö; Foresman, J. B.; Ortiz, J. V.; Cioslowski, J.; Fox, D. J. *Gaussian 09, Revision C.01*, Gaussian, Inc.: Wallingford, CT, **2010**.
5. Yanai, T.; Tew, D. P.; Handy, N. C. *Chem. Phys. Lett.* **2004**, *393*, 51.
